# Comparative analyses of the variation of the transcriptome and proteome of *Rhodobacter sphaeroides* throughout growth

**DOI:** 10.1186/s12864-019-5749-3

**Published:** 2019-05-09

**Authors:** Jochen Bathke, Anne Konzer, Bernhard Remes, Matthew McIntosh, Gabriele Klug

**Affiliations:** 1 Institute of Bioinformatics, University of Giessen, Giessen, Germany; 2 Biomolecular Mass Spectrometry, Max-Planck-Institute for Heart and Lung Research, Bad Nauheim, Germany; 3 Institute of Microbiology and Molecular Biology, University of Giessen, Giessen, Germany

## Abstract

**Background:**

In natural environments, bacteria must frequently cope with extremely scarce nutrients. Most studies focus on bacterial growth in nutrient replete conditions, while less is known about the stationary phase. Here, we are interested in global gene expression throughout all growth phases, including the adjustment to deep stationary phase.

**Results:**

We monitored both the transcriptome and the proteome in cultures of the alphaproteobacterium *Rhodobacter sphaeroides*, beginning with the transition to stationary phase and at different points of the stationary phase and finally during exit from stationary phase (outgrowth) following dilution with fresh medium. Correlation between the transcriptomic and proteomic changes was very low throughout the growth phases. Surprisingly, even in deep stationary phase, the abundance of many proteins continued to adjust, while the transcriptome analysis revealed fewer adjustments. This pattern was reversed during the first 90 min of outgrowth, although this depended upon the duration of the stationary phase. We provide a detailed analysis of proteomic changes based on the clustering of orthologous groups (COGs), and compare these with the transcriptome.

**Conclusions:**

The low correlation between transcriptome and proteome supports the view that post-transcriptional processes play a major role in the adaptation to growth conditions. Our data revealed that many proteins with functions in transcription, energy production and conversion and the metabolism and transport of amino acids, carbohydrates, lipids, and secondary metabolites continually increased in deep stationary phase. Based on these findings, we conclude that the bacterium responds to sudden changes in environmental conditions by a radical and rapid reprogramming of the transcriptome in the first 90 min, while the proteome changes were modest. In response to gradually deteriorating conditions, however, the transcriptome remains mostly at a steady state while the bacterium continues to adjust its proteome. Even long after the population has entered stationary phase, cells are still actively adjusting their proteomes.

**Electronic supplementary material:**

The online version of this article (10.1186/s12864-019-5749-3) contains supplementary material, which is available to authorized users.

## Background

Most bacteria live in environments that do not sustain continuous growth. Rather, natural populations are typically stationary with respect to growth, since factors required for growth are usually limiting (e.g., nutrients, oxygen) or growth-inhibiting factors (e.g., waste products) have accumulated (reviewed in [1]). However, stationary phase is not necessarily the end state. Bacterial growth resumes as soon as environmental conditions become favorable again. Presumably, stationary phase populations are optimized to cope with either of two possibilities. On one hand, they must withstand the challenging environmental conditions that prevent growth. On the other hand, they should be capable of rapidly resuming growth in case of environmental changes that favor growth. A goal of this study was to learn how the bacteria cope with this situation. Transcriptomic and proteomic data on a bacterial population as it traverses through the various growth states can provide some hints. Transcriptomic approaches have been used to study the changes between the growth phases [2, 3]. A combined transcriptomic and proteomic approach has the advantage of providing deeper insights into the molecular changes underlying bacterial growth adaptation [4–7]. Generally, we expected the information flow from DNA to RNA to correlate with the RNA to protein conversion, i.e., changes in the transcriptome are predicted to lead to corresponding changes in the proteome and metabolome. However, evidence is emerging from a wide spectrum of organisms that RNA abundances do not generally correlate well with protein abundances [8]. Another goal of this study was to see if this is indeed the case.

In a previous study, we analyzed transcriptome data from our model organism, *Rhodobacter sphaeroides*, in the exponential phase and after various incubation times in the stationary phase [9]. *R. sphaeroides* is a facultative phototrophic alphaproteobacterium, mostly found in fresh water habitats. These environments are subject to frequent changes, and this is reflected in the flexibility of *R. sphaeroides* to select from a number of metabolic pathways to optimize growth and minimize stress. For example, *R. sphaeroides* uses aerobic respiration for ATP production in the presence of sufficient oxygen levels. Should the oxygen tension decrease, the bacteria start to form photosynthetic complexes. If light is available, anoxygenic photosynthesis is performed [10]. Since the simultaneous presence of light, oxygen and bacteriochlorophyll leads to photooxidative stress by the generation of the harmful singlet oxygen, the bacteria try to avoid this situation by repressing photosynthesis gene expression in the presence of light and oxygen [11, 12]. Additionally, they mount a response to protect against the ensuing photooxidative stress [13, 14]. The response of *R. sphaeroides* to several stresses like oxidative stress (hydrogen peroxide), photooxidative stress (singlet oxygen), and iron limitation has been intensively studied [14–19]. This large body of knowledge on the *R. sphaeroides* response to various stresses provides us with an opportunity to compare the transcriptome of this bacterium in various stress responses with the transcriptome at different growth phases. In particular, we are interested in common regulatory factors and expression patterns.

The success of this approach was exemplified in a previous study on *R. sphaeroides* that focused on transcriptomic changes during exit from stationary phase following access to fresh nutrients (referred to hereafter as outgrowth). Our data revealed that the alternative sigma factors RpoHI and RpoHII are important players in the outgrowth following an extended stationary phase. RpoHI and RpoHII are also required for the response to a variety of stresses, including heat, singlet oxygen, hydrogen peroxide, superoxide, and CdCl_2_ [20]. Remarkably, there was a significant overlap between the outgrowth transcriptome and that of the photooxidative stress response [9], suggesting that the bacterial response to the sudden change in conditions which initiate outgrowth is similar to the photooxidative stress response.

This study presents a comprehensive comparative analysis of changes in the transcriptome and compares this to the proteome over the different growth phases. We include not only transcriptomic and proteomic data during the transition to stationary phase, but also the proteome in deep stationary phase. The transcriptome data are based on a previously published microarray analysis [9], and the proteome data are based on quantitative mass spectrometry.

## Results

### Global comparison of transcriptome and proteome changes during growth

Data from cultures in mid-exponential phase (optical density at 660 nm (OD_660_) 0.5–0.6) served as reference for analyzing data from all other growth phases. These included the transition phase at 10 h after inoculation (OD_660_ 1.2–1.3, trans), initial stationary phase at 28 h (OD_660_ 1.7–1.8, stat 28 h), deep stationary phase at 72 h (OD_660_ 1.1–1.2, stat 72 h), and the following outgrowth from stationary phase upon dilution of stationary phase culture with fresh medium to an OD_660_ of 0.2 [9]. This dilution of stationary phase culture was performed either after 28 h (out 28 h 20’and out 28 h 90′) or after 72 h (out 72 h 20’and out 72 h 90′) of incubation and samples were taken at 20 min and 90 min after dilution. Changes in gene expression were detected by comparing expression levels at later phases to those obtained from exponential phase cultures and are referred to hereafter as log_2_ fold changes (log_2_FC). All expression changes (log_2_FC) are listed in Additional file 1: Table S1.

For the transcripts, RNA of six independent cultures was combined into two pools (three to each pool), and then each pool was hybridized to one array. Thus, the data for each growth phase is based on two microarray data sets, each based on pooled RNA from three independent bacterial cultures. For the proteome analysis, the data for each growth phase is based upon three independent measurements, each representing a pool of extracts from three independent cultures.

**Fig. 1:**
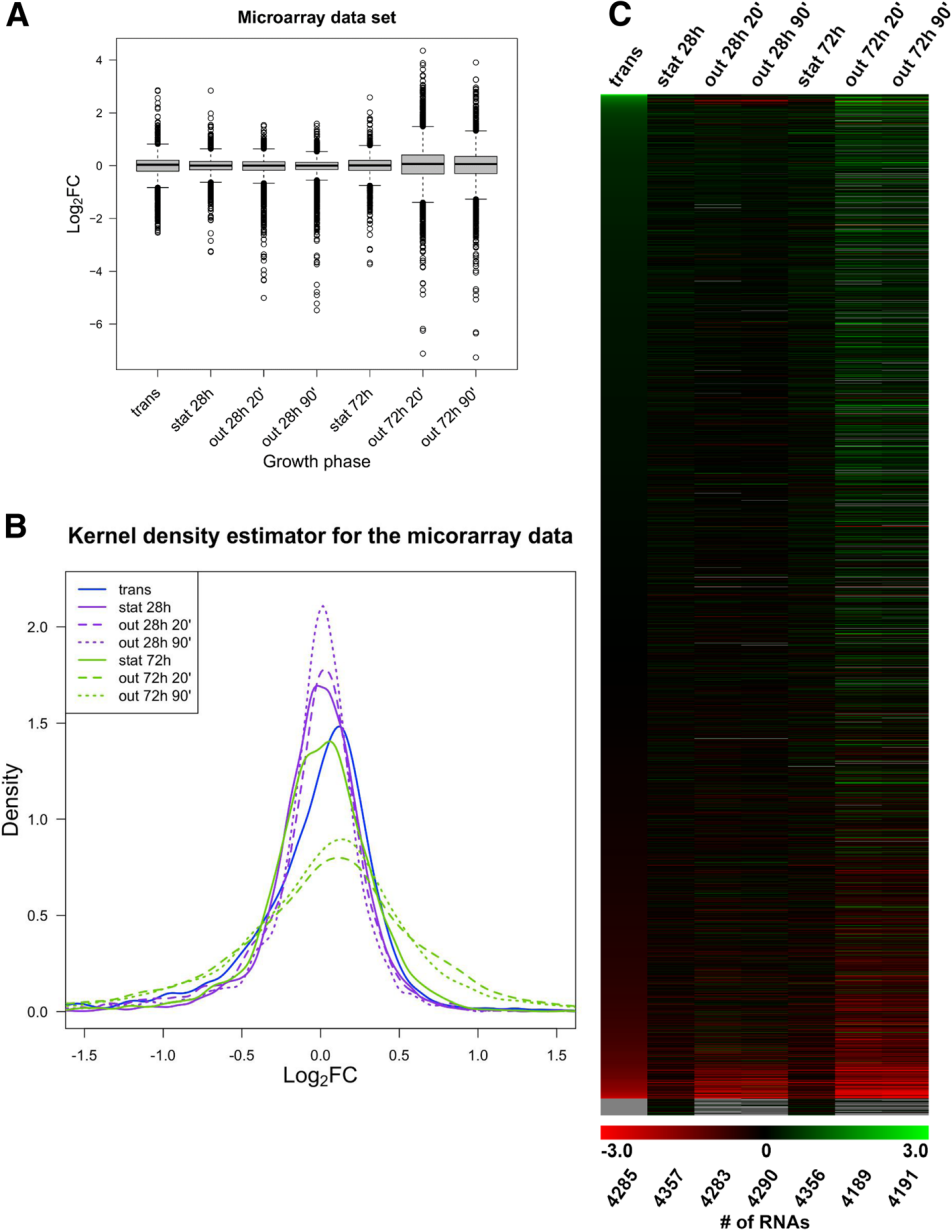
Changes of RNA levels as monitored by microarrays throughout the growth phases. Levels of RNAs in later growth phases were normalized to RNA levels in exponential phase. Box plots (**a**) give an overview on the transcriptome variability of the different samples. Kernel density estimators (KDE) (**b**) compare the distribution of growth phase-dependent changes in gene expression. The heat map (**c**) gives a global overview of the changes in RNA levels at single gene resolution. The ranking of genes is based upon the degree of log_2_FC observed in transition phase, with the highest increases in transcript abundance in the transition phase appearing at the top of the heat map. Indicated underneath the heat map is the total number of transcripts detected at each growth phase

**Fig. 2:**
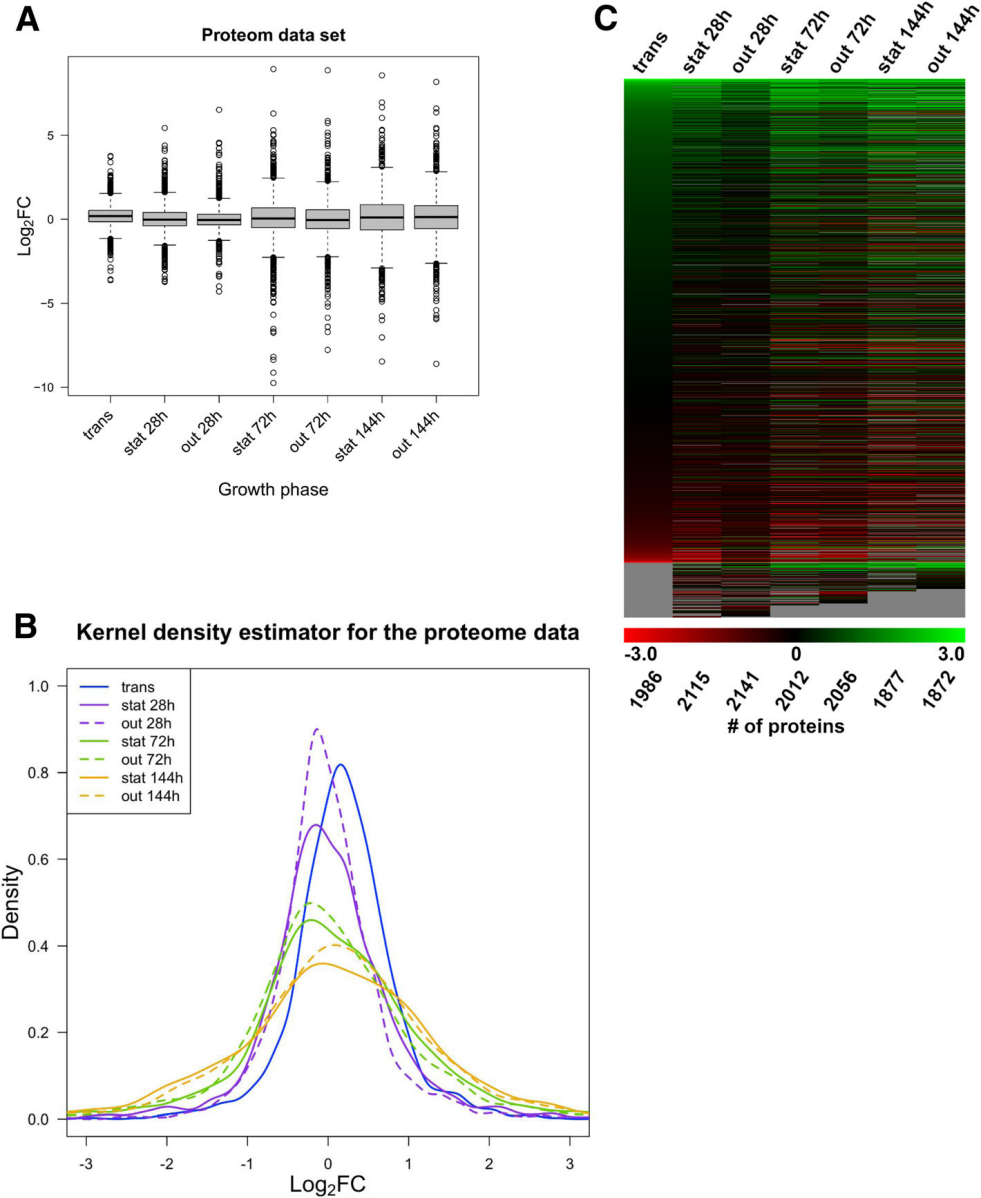
Changes of protein levels as monitored by quantitative mass spectrometry throughout the growth phases. Levels of proteins in later growth phases were normalized to protein levels in the exponential phase. Box plots (**a**) give an overview on the proteome variability of the different samples. Kernel density estimators (KDE) (**b**) compare the distribution of growth phase-dependent changes in protein levels. The heat map (**c**) gives a global overview of the changes in protein levels for each individual gene. The ranking of genes is based upon the degree of log_2_FC observed in transition phase, with the highest increases in protein abundance in the transition phase appearing at the top of the heat map. Indicated underneath the heat map is the total number of proteins detected at each growth phase

Figure 1 depicts the changes in the transcriptome over time and Fig. 2 the changes in the proteome. Box plots (Figs. 1a, 2a) provide a summarized overview of the dynamics of the transcriptome and proteome at different growth phases, where the height of the boxes indicates overall variation in RNA or protein levels. Figure 1a indicates low interquartile distance, i.e., that most transcripts with detectable change show mild changes in expression (log_2_FC < 2) over the various growth phases. Only relatively few transcripts show strongly changed levels. For a more precise description, Additional file 2: Table S2 lists an eight-number summary for each of the transcriptome samples. Additional file 2: Table S2A is essentially a numeric representation of the graphical box plot of Fig. 1a, plus details on the amounts of measured transcripts in each sample. In Fig. 1a, genes detected as having changed transcript levels are represented by circles, with the strongest changes located furthest from zero on the Y-axis. Compared to the data points from the transition phase (trans), where adaption from exponential phase to stationary phase occurs, the following growth phases tend to show stronger changes in transcript abundance. In most cases, the abundance decreases. The extent of most transcript changes is relatively low for early (28 h) stationary phase and subsequent outgrowth as well as for late (72 h) stationary phase. In contrast, outgrowth after 72 h showed a relatively high number of transcripts with very strong changes. A kernel density estimator (KDE) also shows this pattern in Fig. 1b. The KDE models the distribution of the underlying data and is comparable to a smoothened histogram. As the area underneath the curve is always normalized to 1, a direct comparison of distributions, even between samples with different sample sizes, is possible. In the KDE plot (Fig. 1b), a lower peak in the center of the curve, which is always close to zero (on the x-axis), correlates to a higher number of genes with changed transcript levels. Additionally, the broader the peak, the more transcripts show changed level. Thus, the KDE plot reveals that the transcripts from the outgrowth samples after 72 h are very similar to one another and distinct from those of the other growth phases. These outgrowth samples tend to have a higher amount of changed transcripts and fewer transcripts with non-changed levels. Additionally, this pattern is easily observable via a heatmap (Fig. 1c).

A similar analysis was performed for the proteome (Fig. 2 and Additional file 2: Table S2B), with the exception of an additional sample taken 144 h after inoculation and another during the following outgrowth. For protein quantification, the outgrowth samples were taken only at the later time point of 90 min after dilution with fresh nutrients (not at 20 min), since a preliminary proteome analysis revealed very little differences between 20 min and 90 min (data not shown). As was the case for the transcriptome, analysis of the proteomic data revealed that the changes in protein expression generally became more pronounced with increasing culture age. Interquartile distance, whiskers and extremes increased in the later stationary phases (72 h and 144 h) and their respective outgrowths (Fig. 2a). However, the samples taken 144 h after inoculation do not show larger variability than the 72 h samples. Interestingly, the scattering of strongly increased and strongly decreased proteins is considerably more symmetrical than that for the transcriptome, which showed a clear tendency towards a higher number of decreased transcripts. Again, this observation is supported by the KDE analysis (Fig. 2b). Another difference to the transcriptome is the similarity between 72 h stationary phase and following outgrowth (see also the 144 h samples). In the proteomic data, the 72 h stationary phase KDE peak was typically close to its respective outgrowth peak, whereas in the transcriptome data, the 72 h stationary phase peak and its respective outgrowth peak had moved further apart, indicating stronger changes in the transcriptome during outgrowth.

Our data revealed that both transcriptome and proteome show rising variability with increasing culture age, which is reflected by widening box plots and KDEs. Within the KDEs, a shift of density from high peaks in the middle in earlier phases to broader curves in later phases is observable. This widening dynamic reflects the increasing amounts of transcripts or proteins with changed levels. This is further confirmed by the percentages of transcripts/proteins with changed level as discussed in the next section.

Overall, the proteomic data indicated higher dynamic changes than the transcriptome, as judged by comparing the box plots (Figs. 1a and 2a), accompanying 8-number summaries (Additional file 2: Table S2) and heatmaps (Figs. 1c and 2c).

The relative heights of the heatmaps reflect the numbers of detected transcripts or proteins. The heatmap analysis is also useful at this point, since the gray regions at the bottom of the heatmaps represent transcripts and proteins that were undetected in some growth phases. Sample sizes are also provided in the 8-number summaries (Additional file 2: Table S2A, Additional file 2: S2B). The total number of transcripts detected (≈4300) was approximately double the number of proteins (≈ 2000). In a previous study by Remes et al. (2017), the threshold for detection of changed transcripts was set at a log_2_FC of > 0.65 or < − 0.65. We have used this same cutoff, both for the transcriptome and the proteome. The data is summarized in Table 1.

**Table 1:** Comparison between overall changes in RNA transcripts and proteins using a log2FC of > 0.65 or < − 0.65

RNA transcripts (microarray)								
	trans	stat28h	out 28 h 20′	out 28 h 90’	stat 72 h	out 72 h 20′	out 72 h 90’	Sum
Total number	4285	4357	4283	4290	4356	4189	4191	29,951
Total changed log_2_FC (<− 0.65 or > 0.65)	397	175	353	275	265	1117	910	3492
Percentage	9.26%	4.02%	8.24%	6.41%	6.08%	26.67%	21.71%	11.66%
Up (> 0.65)	84	55	56	40	92	583	415	1325
Percentage up	1.96%	1.26%	1.31%	0.93%	2.11%	13.92%	9.90%	4.42%
Down (<−0.65)	313	120	297	235	173	534	495	2167
Percentage down	7.30%	2.75%	6.93%	5.48%	3.97%	12.75%	11.81%	7.24%
Proteins								
	trans	stat 28 h	out 28 h	stat 72 h	out 72	stat 144 h	out 144 h	Sum
Total number	1986	2115	2141	2012	2056	1877	1872	14,059
Total changed log_2_FC (<−0.65 or > 0.65)	471	599	427	886	864	1028	947	5222
Percentage	23.72%	28.32%	19.94%	44.04%	42.02%	54.77%	50.59%	37.14%
Up (> 0.65)	346	318	212	500	436	579	544	2935
Percentage up	17.42%	15.04%	9.90%	24.85%	21.21%	30.85%	29.06%	20.88%
Down (<−0.65)	125	281	215	386	428	449	403	2287
Percentage down	6.29%	13.29%	10.04%	19.18%	20.82%	23.92%	21.53%	16.27%

**Fig. 3:**
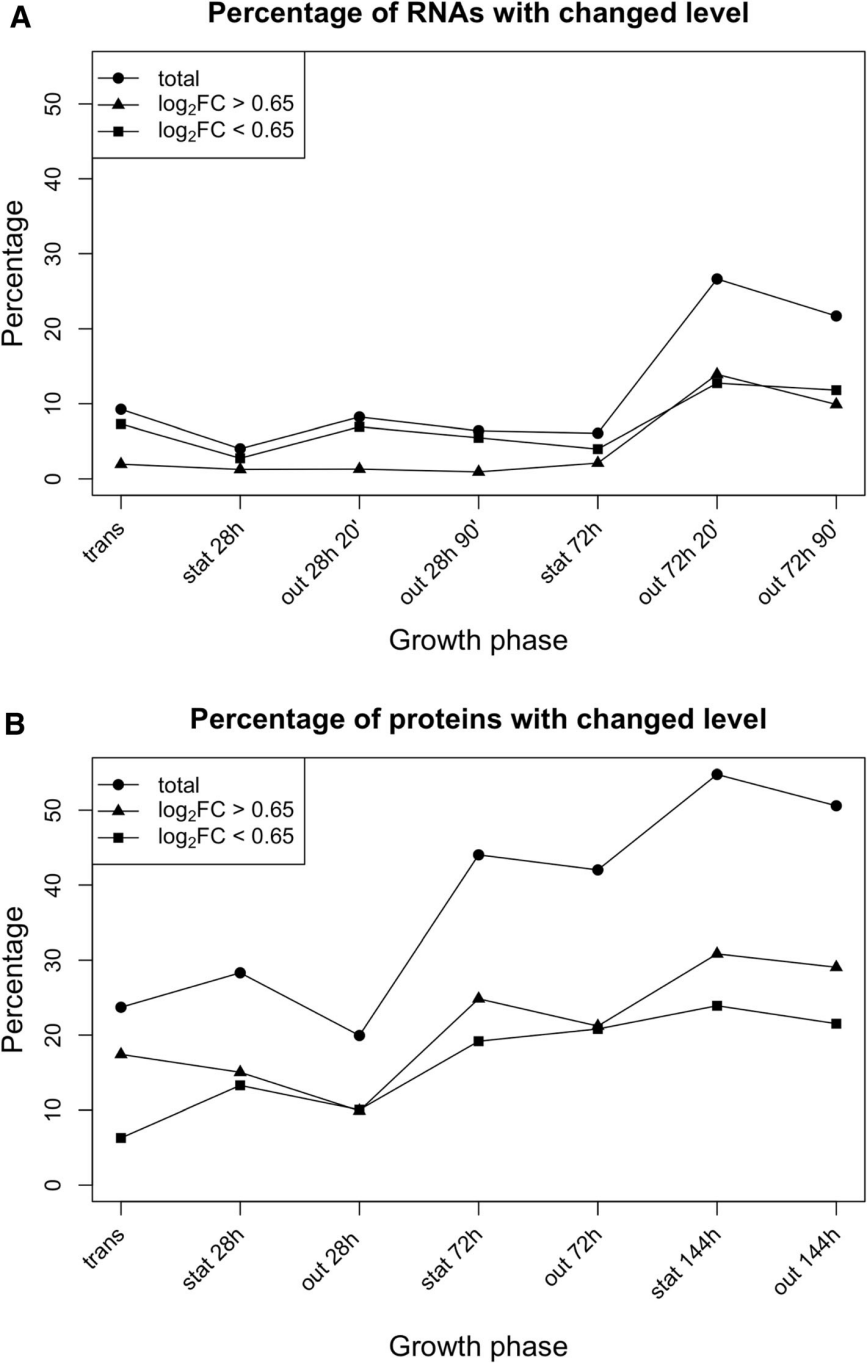
Percentage of transcripts (**a**) and proteins (**b**) with changed levels throughout growth. For both the microarray and proteome data, the status of changed versus not-changed were determined using thresholds of log_2_FC 0.65 and − 0.65. In each case, the percentage of transcripts or proteins with levels exceeding the cutoffs were determined for each growth phase. For a direct comparison, both graphs harbor identical scales on the ordinate

Using the threshold of log_2_FC of > 0.65 or < − 0.65, the number of proteins detected as changed (compared to levels in the exponential phase) were typically higher than the number of transcripts detected as changed. In fact, the sum total of changed proteins over all growth phases averaged at 37% of all detected proteins, while that of changed transcripts only at 11%. This difference is illustrated in Fig. 3. We interpret this outcome to reflect the higher dynamic range of changes on the proteome level. Also noteworthy is that the percentage of proteins with changed levels rises in the stationary phase, particularly in the extended stationary phase (72 h and 144 h) and the following outgrowth. In contrast, the percentage of transcripts with changed levels remains relatively low (< 10%) in all growth phases except for the outgrowth following deep stationary phase (72 h).

### Correlation of individual growth phases

The pattern of transcripts and proteins with changed levels within each growth phase was addressed in the previous section. How does the pattern compare between the growth phases? To get an in-depth view of the similarity between all growth phases, Pearson correlation coefficients (PCC) were generated for all possible combinations using the microarray data as well as the proteome data and presented as dendrograms in the supplementary data Additional file 5: Figure S1. The most important comparisons are listed in Table 2A and B. As expected, high degrees of correlation for transcriptome samples were observed between stationary phases at 28 and 72 h (PCC of 0.90). This fits to a scenario in which the transcriptome is relatively static upon reaching the stationary phase. Also, the outgrowth samples revealed high correlation between 20 min and 90 min, regardless of whether this outgrowth followed early (28 h, PCC of 0.92) or extended (72 h, PCC of 0.96) stationary phase. In contrast, correlation was lower when comparing outgrowths from early versus extended stationary phase (PCCs between 0.41 and 0.53). This fits with a previous observation that the duration of the stationary phase has a strong effect on the outgrowth transcriptome [9]. The Remes et al. study also showed that the outgrowth has a dramatic effect on the transcriptome. That effect is also indicated here by the low PCC values of the microarray samples (stat 28 – out 28 h 20′: 0.345 and stat 28 h – out 28 h 90′: 0.339). We observe a similar situation for the outgrowth after deep stationary phase (stat 72 h – out 72 h 20′: 0.44 and stat 72 h – out 72 h 90′: 0.406). Hence, the transcriptome appears static during stationary phase, and undergoes radical remodeling during outgrowth.

**Table 2:** Correlation of microarray data for different growth phases

Comparison	PCC	Comparison	PCC	Comparison	PCC
A					
trans – stat 28 h	0.503	stat 28 h – stat 72 h	0.902	stat 28 h – out 28 h 20’	0.345
trans – out 28 h 20’	0.518	out 28 h 20′ – out 28 h 90’	0.915	stat 28 h – out 28 h 90’	0.339
trans – out 28 h 90’	0.485	out 28 h 20′ – out 72 h 20’	0.457	stat 72 h – out 72 h 20’	0.440
trans – stat 72 h	0.443	out 28 h 20′ – out 72 h 90’	0.529	stat 72 h – out 72 h 90’	0.406
trans – out 72 h 20’	0.622	out 28 h 90′ – out 72 h 20’	0.414		
trans – out 72 h 90’	0.642	out 28 h 90′ – out 72 h 90’	0.487		
		out 72 h 20′ – out 72 h 90’	0.958		
B					
trans – stat 28 h	0.760	stat 28 h – stat 72 h	0.724	stat 28 h – out 28 h	0.835
trans – out 28 h	0.650	stat 28 h – stat 144 h	0.578	stat 72 h – out 72 h	0.941
trans – stat 72 h	0.527	stat 72 h – stat 144 h	0.864	stat 144 h – out 144 h	0.977
trans – out 72 h	0.493	out 28 h – out 72 h	0.721		
trans – stat 144 h	0.411	out 28 h – out 144 h	0.590		
trans – out 144 h	0.412	out 72 h – out 144 h	0.841		

The situation is strikingly different on the proteome level. There is a high degree of correlation between stationary and outgrowth phase at 28 h (PCC of 0.83). Especially later measurements at 72 and 144 h show an extremely high degree of correlation between the respective stationary and outgrowth phase with PCCs of 0.94 and 0.98 respectively. Also in contrast to the transcriptome, the proteome shows more variation throughout the stationary phase. Rather than stasis, the proteome continues to change between early and late stationary phase (stat 28 h-stat 72 h: 0.72), compared to a value of 0.90 at the transcriptome level. Thus, the proteome is more likely than the transcriptome to continue changing throughout the stationary phase, and less likely to change during the outgrowth. Despite this difference, both the transcriptome and the proteome show a similar trend: the length of the stationary phase has a strong impact on not only the outgrowth transcriptome [9] but also the outgrowth proteome. The dendrograms (Additional file 5: Figure S1A & S1B) provide an overview of these findings.

For a direct comparison of each transcript with its encoded protein at distinct growth phases, scatter plots and accompanying heatmaps were generated (supplementary data Additional file 6: Figure S2 A-K). Comparisons at outgrowth phases at 20 min was omitted since the transcriptome at this time point was highly similar to that at 90 min. The scatter plots and heatmaps clearly reveal the generally high level of discontinuity between transcript and protein. For example, rather than showing that transcripts with changed levels tend to have a correspondingly altered protein level, the data points appear evenly scattered. Also noticeable is the considerably broader distribution of data points on the ordinate compared to the abscissa. As we have previously observed from the box plot analysis (Figs. 1 and 2), this reflects the wider dynamics of the proteome compared to the transcriptome. The lack of correlation between RNA and protein levels is also reflected by PCCs for the scatter plots (Table 3, see also Additional file 5: Figure S1C for a heatmap). Even the highest correlation value of 0.25 observed at outgrowth following stationary phase at 72 h (out 72 h 90′-out 72 h) can be consider uncorrelated. Since the PCC value did not indicate any linear correlation, Spearman’s rank correlation coefficient (indicating monotonic correlations) was also determined. However, no monotonic correlation was observable, as can be seen from the low values. We also considered the possibility that changes in the transcriptome are likely to correspond to delayed changes in the proteome, due to the time needed for translation of transcript into protein. Therefore, we also compared the transcriptome at transition phase to the proteome in early stationary phase (28 h), and the early stationary phase transcriptome to the proteome at 72 h. Again, correlation of the data sets was very low. This result contrasts with that of an earlier study, in which we observed a much better correlation (Pearson correlation *r* = 0.64) between transcriptomic and proteomic changes 90 min after initiating photooxidative stress [14]. The significance of the higher correlation during photooxidative stress is that this demonstrates that the *R. sphaeroides* proteome has a potential for rapid remodeling (and thus high correlation between the transcriptome and proteome). Why this is not the case here, during the transition from exponential to stationary phases, is currently unknown.

**Table 3:** Correlation coefficients for comparisons of changes in RNA and protein levels

	Comparison	Pearson	Spearman	# of data points
01	trans – trans	0.025	0.084	1973
02	trans – stat 28 h	0.189	0.175	2101
03	stat 28 h – stat 28 h	0.121	0.131	2105
04	stat 28 h – stat 72 h	0.199	0.205	2002
05	stat 28 h – stat 144 h	0.162	0.140	1869
06	out 28 h 90′ – out 28 h	−0.048	−0.178	2127
07	out 28 h 90′ – out 72 h	−0.032	−0.231	2044
08	stat 72 h – stat 72 h	0.147	0.153	2002
09	stat 72 h – stat 144 h	0.110	0.096	1869
10	out 72 h 90′ – out 72 h	0.245	0.222	2039
11	out 72 h 90′ – out 144 h	0.182	0.163	1859

The low correlation between transcriptome and proteome indicate that changes in the proteome throughout growth are mostly not solely the consequence of changes in the transcriptome. We believe that post-transcriptional regulation likely plays a major role in determining protein accumulation under these conditions. Important candidates of post-transcriptional regulation are the small non-coding RNAs (sRNAs). Changes in sRNA levels are not included in the diagrams shown in this study since they cannot be directly correlated to a protein product. However, as already described in Remes et al. (2017) and also shown in Additional file 3: Table S3, several sRNAs show altered levels in later stages of growth or during outgrowth. For example, 18 sRNAs showed an increase of > 0.65 log_2_FC (log_2_FC > 5 for IGR_0827) in the outgrowth following 72 h stationary phase, while 46 sRNAs decreased by log_2_FC < − 0.65 (log_2_FC < − 4.75 for IGR_2249) (see Additional file 3: Table S3). Altogether more than half of the sRNAs detected in the microarray analysis showed changed expression in outgrowth after 72 h stationary phase and only 22% showed growth phase-independent expression.

### Changes of the proteome throughout growth phases

While changes in the transcriptome during growth have already been analyzed in detail [9], this is not the case for the proteome. The total number of proteins detected in each growth phase, together with the subsets of proteins with increased and decreased levels, are listed in Table 1.

**Fig. 4:**
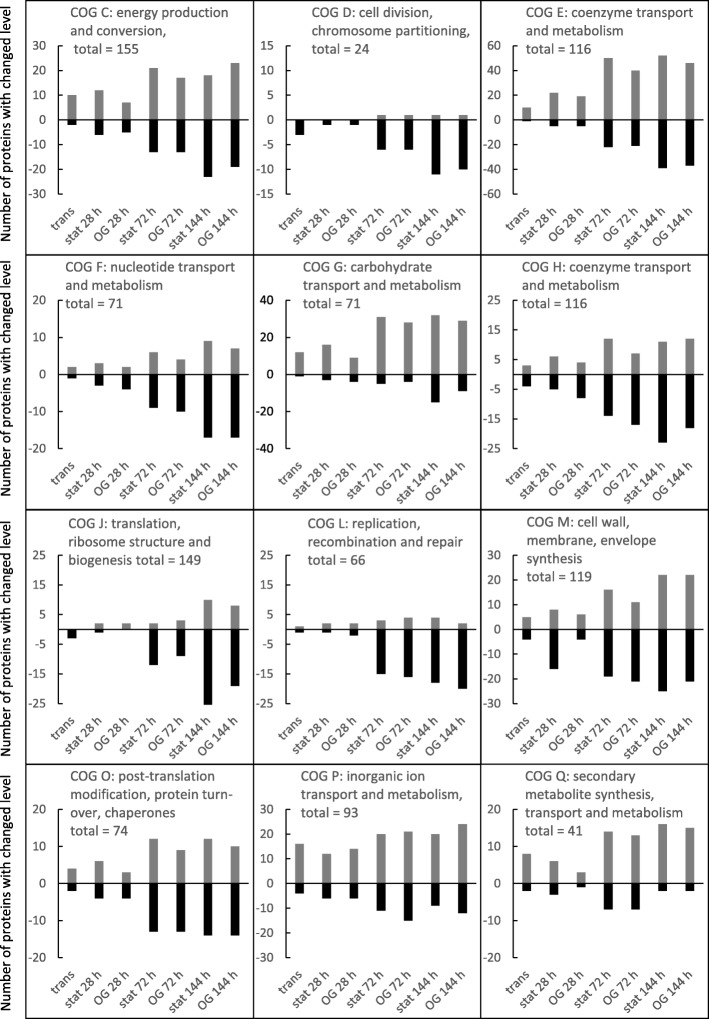
Number of proteins with decreased levels (represented as negative values on the Y-axis) or increased levels (represented as positive values on the Y-axis) throughout growth in selected COGs, determined using thresholds of log_2_FC 0.65 and − 0.65. The complete data set is provided in Additional file 4: Table S4

Figure 4 provides an overview of the number of proteins belonging to selected clusters of orthologous groups (COGs) whose accumulations change with the different stages of growth relative to the total number of proteins detected within each COG. The complete data set for the individual proteins assigned to the different COGs are shown in Additional file 4: Table S4. Some COGs were not included in our analysis. For example, none of the *R. sphaeroides* proteins are assigned to COG-A (RNA processing and modification), COG-B (chromatin structure and dynamics), COG-W (extracellular structures), COG-Y (nuclear structure), or COG-Z (cytoskeleton). Furthermore, a number of *R. sphaeroides* proteins in COGs R and S have no known function, while the total number of proteins and the number of changes in COGs-N (cell motility), U (intracellular trafficking), and V (defense mechanisms) was too low for a meaningful analysis.

Compared to the exponential proteome, the stationary phase proteome appeared to differ significantly.

Within each COG (Fig. 4), the number of proteins with increased levels (indicated as positive on the Y-axis) or decreased levels (indicated as negative on the Y-axis) changed with culture age. For example, in COG-Q (secondary metabolite synthesis, transport and metabolism), levels of 14 proteins (from a total of 41 detected proteins) increased in the stationary phase (72 h) compared to the exponential phase. The complete data set is provided in Additional file 4: Table S4. Almost 26% of all detected proteins in COG-C (energy production and conversion) showed either higher (18 from 155) or lower levels (22 from 155) after 144 h of incubation compared to the exponential phase. In COG-J (translation ribosome structure and biogenesis), the largest change also occurred after 144 h of incubation, but this involved only 24% of all proteins, most of which showed decreased levels. Altogether, this data suggests a highly dynamic proteome during the transition from exponential growth to stationary phase, as noted above. Also noteworthy is the observation that some COGs contained proteins that continued to change after 72 h. Even at 144 h, protein levels continued to change. Many of the changes between 72 h and 144 h involved protein decreases, indicating protein degradation, which we expect to be prevalent in the stationary phase. However, others, such as COG-K (transcription), COG-M (cell wall, membrane, envelope synthesis), COG-O (post-translational modification and protein turn-over), COG-T (signal transduction), showed a similar number of increased and decrease proteins in the deep stationary phase (144 h, Fig. 4). These data indicate that signal transduction and transcriptional regulation are still very active in the late growth phases, that synthesis of cell material is not necessarily lower than in the exponential phase, and that proteins involved in protein turn-over are equally increased and decreased.

A generally higher number of increased proteins in the stationary phase (compared to the exponential phase) was observed for the categories COG-C (energy production and conversion), COG-E (amino acid transport and metabolism), COG-G (carbohydrate transport and metabolism), COG-I (lipid transport and metabolism), COG-P (inorganic ion transport and metabolism), and COG-Q (secondary metabolite synthesis, transport and metabolism) (Fig. 4). This most likely reflects the attempt to guarantee the supply of nutrients and building blocks when their availability in the surrounding medium is dwindling. The number of increased proteins in COG-G and COG-I is particularly higher than the number of decreased proteins at all stages of growth.

A generally higher number of decreased proteins in the stationary phase was observed for the categories COG-D (cell division, chromosome partitioning), COG-F (nucleotide transport and metabolism), COG-H (Coenzyme transport and metabolism), and COG-L (replication, recombination, repair). These patterns match well to a lower demand for proteins required for cell division upon entry into stationary phase. Also decreasing in the stationary phase were the proteins in COG-J (translation, ribosome structure and biogenesis, Fig. 4). Although most (75%) of the COG-J proteins showed unchanged levels, of those which showed change, the majority decreased. We are unsure of the significance of this result. Considering that protein production is apparently very active in the stationary phase, it is unclear why proteins related to translation, ribosome structure and biogenesis show a decreasing trend.

We have provided here a COG-based overview of the changes in the proteome throughout the growth phases. A detailed description of specific protein changes throughout the different stages of growth is provided in the supplementary material.

## Discussion

The goal of this study was to monitor and to compare the adaptation of a bacterial model organism throughout different growth phases at both the transcriptome and proteome levels. Our data revealed a much higher dynamic within the proteome compared to the transcriptome. We cannot exclude that this higher dynamic is at least partly due to the different methods used for the respective transcriptomic and proteomic samples. For example, detection efficiencies and sensitivity limits for each method are not necessarily identical. Some difference between the transcriptome and proteome might be due to an extremely low abundance of transcripts or proteins or, in the case of protein, poor recovery due to low solubility or membrane attachment. Other explanations for the different distribution of changed transcripts versus changed protein levels over the different phases of growth include the fact that a single transcript can be translated multiple times into protein, and that proteins are frequently more stable than transcripts, meaning that proteins are more likely to accumulate than transcripts. Especially during outgrowth, some transcripts may decrease due to rapid degradation, while proteins produced in the stationary phase are more stable and are therefore detectable in the following outgrowth despite the lack of production in that phase of growth.

Another likely explanation for the transcriptome and proteome differences is post-transcriptional regulation. The weak correlation between the transcriptome and the proteome implies a major role for regulation at the post-transcription level in growth phase adaptation. Important roles for sRNAs in growth phase adaptation have been described [21–24]. sRNAs mostly affect gene expression by altering the stability of mRNAs or by promoting or inhibiting translation (reviewed in [25]). It is also conceivable that transcript stability may change at different growth phases [26]. Presently we know very little about growth phase-dependent processing and degradation of RNA. Variation in the translation rates of mRNAs may also vary in different growth phases independently of the action of sRNAs. This also applies to proteolysis rates, and may impact the accumulation of proteins in different growth phases [27].

The lack of correlation between transcriptome and proteome data also implies that changes of the transcriptome mostly lead to minimal or transient changes of the proteome that are not manifested over longer time intervals. Earlier studies on growth phase-dependent changes in RNA and protein levels have noted such weak correlation between the data sets [5, 7, 28] and raised the question of to what extent is the bacterial proteome regulated at the level of transcription [6].

Our analysis of the transcriptome and proteome has revealed a number of distinct characteristics of global gene expression in *R. sphaeroides*. We suggest that these differences reflect a survival strategy of *R. sphaeroides* in ensuring a rapid and robust response to environmental changes. Although the number of detected proteins was only half that of the transcripts, variation in protein accumulation during the transition to stationary phase was more dynamic. Apparently, the bacterium responds to gradually deteriorating environmental conditions mostly at the protein level. In fact, the proteome continued to change in the extended stationary phase, even into deep stationary phase, while the transcriptome remained mostly static. Possibly, further changes in the transcriptome during stationary phase are not necessary, since the transcripts that are needed to deal with suboptimal conditions have been already produced during exponential growth. This is not unlikely, since bacteria prepared for hostile conditions would enjoy a significant survival advantage over unprepared ones, given that unfavorable growth conditions usually follow. Hence, post-transcriptional regulation is mostly sufficient to organize the proteome as the bacterium ceases growth and gradually enters stationary phase. Interestingly, in stationary phase, we observed that changes in protein levels continued. This is remarkable, particularly for deep stationary phase (144 h), since that environment has almost ceased to change. How are these proteins regulated in the absence of environmental stimuli? We note that knowledge of gene regulation and bacterial behavior in deep stationary phase is generally lacking, although some progress on the molecular basis of bacterial longevity is being made [29].

Another finding from this study was the marked difference between the transcriptome and proteome upon outgrowth. Changes in the transcriptome were modest during the transition to stationary phase, and especially throughout the extended stationary phase. Upon outgrowth, however, the transcriptome was subject to radical remodeling, most of which occurred within the first 20 min. In contrast, while proteome had the capacity for a greater dynamic range, it was less likely to change during the first 90 min of outgrowth. Hence, the first response to significant changes in the environment is a rapid remodeling of the transcriptome, analogous to the stress response upon photooxidative stress [9]. Higher correlation (0.64) between the proteome and transcriptome was also observed upon oxidative stress [14], implying that these have at least the capacity to show correlation under specific circumstances. Why this does not occur in the first 90 min of outgrowth is unknown.

Despite the above-mentioned differences between the transcriptome and proteome, they show a similar trend: both are strongly impacted by the length of the stationary phase. This observation supports the idea that bacteria continue to change and adjust their physiological state, even well after entering the stationary phase. Presumably, the success of bacterial survival depends upon their ability to survive suboptimal conditions on one hand, and rapidly resume bacterial growth in the case that environmental conditions turn favorable. Bacteria apparently resolve this problem by using complex regulation at both transcriptional and post-transcriptional levels. Further work is needed to understand how the bacteria organize and maintain their proteome in the stationary phase.

## Methods

### Strains and growth conditions

*R. sphaeroides* wild type strain 2.4.1(DSMZ 158) was grown at 32 °C in a defined minimal medium with malate as the carbon source [19]. Erlenmeyer flasks were filled to 80% capacity with the culture medium and continuously shaken at 140 rpm, resulting in 25–30 μM dissolved oxygen in exponential phase. The growth curve and oxygen concentration in the cultures are shown in Remes et al., 2017. RNA isolation and microarray analysis are also described in Remes et al., 2017. The microarray data are available at the NCBI Gene Expression Omnibus database (50) under accession number GSE75345.

### Protein sample preparation and mass spectrometry

Pelleted cells were harvested from liquid cultures, lysed in SDS buffer (4% SDS in 0.1 M Tris/HCl, pH 7.6), heated at 70 °C for 5 min and sonicated to shear the DNA. Cell debris was removed by centrifugation at 16000 x g for 10 min prior to estimation of protein concentration by the DC protein assay (BioRad). Solubilized proteins were precipitated by 4 volumes of acetone at − 20 °C for 1 h, pelleted at 14000 x g for 10 min and washed with 90% acetone. Samples were dried to remove acetone completely and dissolved in urea buffer (6 M urea, 2 M thiourea, 10 mM HEPES, pH 8.0). Enzymatic fragmentation of proteins was performed by in-solution digestion [30]. Briefly, protein disulfide bonds were reduced with 1 mM dithiothreitol and alkylated with 5.5 mM iodoacetamide. Next, proteins were cleaved enzymatically by Lys-C (protein to enzyme ratio 100:1) (Wako Chemicals GmbH) at room temperature for 3 h prior to diluting samples to 2 M urea/thiourea by adding 50 mM ammonium bicarbonate. Proteins were further digested by trypsin (protein to enzyme ratio 100:1) (Promega) at room temperature overnight and the resulting mixture of peptides was desalted and concentrated by stop and go extraction (STAGE) tips [31].

### Data analysis

Annotation of clusters of orthologous groups (COG) was based upon the most current version available at time of writing (2003 COGs, 2014 update, available at: http://www.ncbi.nlm.nih.gov/COG/ [32]. To match COG functional annotations and RSP numbers respective data were obtained from UniProt for the organism „*Rhodobacter sphaeroides* (strain ATCC 17023 / 2.4.1 / NCIB 8253 / DSM 158) “(Taxon identifier: 272943) [33]. UniProt data were adjusted to contain columns for RSP and COG numbers. The latter were included in the eggNOG annotation column. Using those data a matching of RSP numbers to COG numbers and finally COG functional annotation was archived. UniProt data were obtained on 30th of November 2017 (available as supplementary data: uniprot-proteome%3A%28taxonomy%3A%22Rhodobacter + sphaeroides+%28strain + ATCC+ 17023--.tab.gz).

Data analysis was performed using R (version 3.4.3), bash (version 3.2.57) and LibreOffice (version 5.3.7.2). Heatmaps comparing growth phases of transcriptome and proteome data were generated using MeV (MultiExperiment viewer - version 4.8.1) [34]. Heatmaps correlating multiple growth phases were generated applying the R package pheatmap. Pearson’s and Spearman’s correlation coefficients were calculated using the R function cor. Heatmaps displaying the variability of COG functional groups according to growth phase were generated using the R package ggplot2 [35]. Further graphics were generated using R integrated graphical functionalities, including the boxplot function and density function (defaulting to a Gaussian density) for drawing the kernel density estimation.

## Additional files

Additional file 1: Summary of all changes in transcriptome and proteome. (XLSX 981 kb)

Additional file 2: Supplementary material. (DOCX 47 kb)

Additional file 3: RNAs showing changes in accumulation at various growth phases. (XLSX 21 kb)

Additional file 4: Proteins showing changed levels at various growth phases and their COG associations. (XLSX 595 kb)

Additional file 5: **Figure S1**. Heatmaps of correlations between transcriptome and proteome. (PPTX 317 kb)

Additional file 6: **Figures S2A-K**. Scatter plots of transcriptome versus proteome throughout growth phases. (PPTX 1040 kb)

## Abbreviations

#: Number
ATP: Adenosine triphosphate
COG: Clusters of orthologous groups
h: Hour
IGR: Intergenic region
KDE: Kernel density estimator
MeV: MultiExperiment viewer
OD: Optical density
out: Outgrowth
PCC: Pearson correlation coefficients
RSP: *Rhodobacter sphaeroides* gene
STAGE: Stop and go extraction
stat: Stationary phase
trans: Transition phase
