# Supplementary material for: Comparative analyses of the variation of the transcriptome and proteome of Rhodobacter sphaeroides throughout growth
Source: BMC Genomics. 2019 May 9;20:358. doi: 10.1186/s12864-019-5749-3 (PMC6509803; doi:10.1186/s12864-019-5749-3)
Supplement: Supplementary file 2 — Supplementary material. (DOCX 47 kb) [file 12864_2019_5749_MOESM2_ESM.docx]

Supplementary File

Table S2A: Eight-number summary for microarray data.

|  | **trans** | **stat28h** | **out 28h 20’** | **out 28h 90’** | **stat 72h** | **out 72h 20’** | **out 72h 90’** |
| --- | --- | --- | --- | --- | --- | --- | --- |
| **Sample size** | 4285 | 4357 | 4283 | 4290 | 4356 | 4189 | 4191 |
| **Median** | 0.03430 | 0.00410 | 0.00310 | 0.00120 | 0.00985 | 0.06630 | 0.06030 |
| **Mean** | -0.04188 | -0.01173 | -0.07385 | -0.06562 | -0.01152 | 0.02166 | -0.00657 |
| **Standard deviation** | 0.4493998 | 0.3229758 | 0.4513647 | 0.4367091 | 0.3833836 | 0.8047981 | 0.7375894 |
| **Minimum** | -2.53180 | -3.26530 | -5.00900 | -5.47830 | -3.72780 | -7.12420 | -7.26840 |
| **Maximum** | 2.86160 | 2.84730 | 1.54430 | 1.59270 | 2.58970 | 4.35160 | 3.90240 |
| **1. quartile** | -0.21110 | -0.15670 | -0.17765 | -0.14538 | -0.18263 | -0.31610 | -0.30005 |
| **3. quartile** | 0.20270 | 0.16120 | 0.14805 | 0.12587 | 0.19703 | 0.40510 | 0.34915 |

Table S2B: Eight-number summary for proteome data.

|  | **trans** | **stat 28h** | **out 28h** | **stat 72h** | **out 72** | **stat 144h** | **out 144h** |
| --- | --- | --- | --- | --- | --- | --- | --- |
| **Sample size** | 1986 | 2115 | 2141 | 2012 | 2056 | 1877 | 1872 |
| **Median** | 0.1824 | -0.02910 | -0.04920 | 0.04235 | -0.05075 | 0.1041 | 0.1214 |
| **Mean** | 0.1931 | 0.01932 | 0.00106 | 0.05662 | -0.01114 | 0.0922 | 0.1195 |
| **Standard deviation** | 0.64112 | 0.80792 | 0.69269 | 1.28670 | 1.17602 | 1.40185 | 1.31224 |
| **Minimum** | -3.6230 | -3.72270 | -4.29440 | -9.74620 | -7.77310 | -8.4623 | -8.6010 |
| **Maximum** | 3.7643 | 5.43500 | 6.51850 | 8.92120 | 8.85190 | 8.5481 | 8.1578 |
| **1. quartile** | -0.1492 | -0.39240 | -0.33720 | -0.50225 | -0.55982 | -0.6375 | -0.5638 |
| **3. quartile** | 0.5261 | 0.40505 | 0.29560 | 0.67645 | 0.56188 | 0.8613 | 0.8023 |

**Changes of the proteome in transition phase**

During the transition to stationary phase (trans), a total of 471 proteins were detected as changed by a log_2_ fold of ≥ 0.65 compared to their respective levels in the exponential phase. 346 proteins showed a log_2_ fold increase ≥ 0.65. Of these, only 146 proteins increased by ≥ log_2_ fold 2. 125 proteins showed a log_2_ fold decrease of ≥ 0.65 (63 proteins decreased by ≥ log_2_ fold 2). Most of the proteins detected as changed in the transition phase retained their status in later growth stages. A small subset of 49 proteins showed an increase that was specific to this phase (≥ log_2_ 2 fold lower in all other growth phases). These proteins belong to various clusters of orthologous groups (COGs) with roles related to metabolic enzymes involved in glycolysis or fatty acid synthesis (Fig. 4, Table S4). Also in this group were multiple transcription factors and the endonuclease III. None of them belonged to the COG D (cell division, chromosome partitioning) or COG N (motility). In fact, none of the COG N proteins were detected throughout the proteome analysis.

The 130 downregulated proteins of the transition phase belong to COG C (energy production and conversion), COG E (amino acid transport and metabolism), COG G (carbohydrate transport and metabolism), COG I (lipid transport and metabolism), (inorganic iron transport and metabolism), COG M (cell wall/membrane/envelope synthesis) and COG K (transcriptional regulators). The levels of many of these continued to fall in the stationary phase (Fig. 4). COG H (coenzyme transport and metabolism) contained the highest number of decreased proteins (9% of all decreased proteins, 3% of the increased proteins). Several proteins involved in bacteriochlorophyll or carotenoid synthesis fall into this category. Downregulation was also the general trend in COG J (translation, ribosomal function, and biogenesis; 6% of decreased proteins, 2.5% of increased proteins) (Fig. 4). In contrast, proteins of COG E (amino acid transport and metabolism, 6% of the decreased proteins, 11% of increased proteins) and COG G (carbohydrate transport and metabolism; 2% of the decreased proteins, 7.9 % of the increased proteins) tended to increase in the transition phase (Fig. 4 and Table S1). This raises the question of what the advantage could be with increasing proteins necessary for amino acid and carbohydrate metabolism and transport precisely at the point when these are depleted or have reached very low levels in the growth medium. One possibility is that such proteins could be involved in scavenging. In such a scenario, detection of scavenging proteins exclusive to the phase of growth at which they are most useful is expected. Interestingly, the same pattern of increased levels of proteins involved in transport and carbohydrate metabolism upon carbon starvation was observed in another member of the *Rhodobacteraceae*, the aerobic anoxygenic phototroph *Roseobacter litoralis* (1).

Increased accumulation specific to the transition phase was also observed for ChrR. This matches our expectations, since under normal growth conditions, ChrR is an anti-sigma factor for RpoE (2), and is proteolytically degraded upon singlet oxygen stress (3). RpoE is a major activator of the singlet oxygen response in *R. sphaeroides* (4-6). RpoE was not detected in our proteome analysis. As with most other proteins with increased levels in transition phase, the *chrR* transcript level did not correlate with its protein level. Instead, increased levels of *chrR* mRNA were only observed during outgrowth after a long stationary phase (72h).

Many proteins showed a clear decrease in the transition phase. These include precorrin-6A synthase (RSP_2819, CobF), and cobaltochelatase (RSP_2827, CobN) of the cobalamin (also known as vitamin B12) synthesis pathway. CobN is the protein with the strongest decrease in the transition phase (log_2_ fold change -3.62). Except for CobK, whose levels slightly increased during transition and even further in the stationary phase, most other detected proteins in this pathway show reduced levels in transition phase (CobW: log_2_ fold change -0.83; CobM log_2_ fold change -3.57; CobF: -1.16) and were either undetected or decreased further in the stationary phase. Altogether, this pattern fits well with the maximal synthesis and accumulation of bacteriochlorophyll, which requires cobalamin synthesis, in the exponential phase (7, 8). In contrast to the cobalamin synthesis proteins, their respective transcripts were unchanged or slightly decreased in the transition phase, reinforcing our conclusion above that the transcriptome and proteome are generally poorly correlated. A lack of correlation was even more obvious with the *sitABCD* genes. The Sit proteins form subunits of an ABC Mn^2+^/Fe^2+^ transporter. While the *sitABCD* transcripts increased strongly in the transition phase, SitA and SitB (RSP_0904 and 0905) showed a decrease specific to the transition phase, and then increased again in the stationary phase. (The remaining subunits, SitC and SitD, were not detected in the proteome analysis.)

Many other proteins showed a clear decrease (by log_2_ fold change ≥ 0.65) in the transition phase but not in their respective transcript levels. These include a catalase (RSP_2779, CatA, formerly KatE), a bacterioferritin (RSP_3342, Bfr), cytochrome b (RSP_1395, FbcB), S-formyl glutathione hydrolase (RSP_0852, FghA), thioredoxin reductase (RSP_1576, TrxB), a putative polysaccharide deacetylase (RSP_1415), a cell wall hydrolase (RSP_1894), a putative metallopeptidase (RSP_2974), an oligoketide cyclase/lipid transport protein (RSP_2787), and an NADPH-dependent FMN reductase (RSP_3069). A second bacterioferritin of *R. sphaeroides* (RSP_1546) also showed a strong decrease in the transition phase (log_2_ fold change -1.25) which persisted into the early stationary phase (log_2_ fold changes of -0.7). It is currently unclear what role bacterioferritin plays in the lifestyle of bacteria. It is conceivable that reduced bacterioferritin levels will help to make iron available for the cell during later growth phases, when metals may become limiting. The decrease in catalase (CatA, decreased 2-fold in the transition phase) persisted into early stationary phase and then increased in all later growth phases. This may be related to the oxygen levels, which drop in the exponential phase, remain low in the transition phase and then increase in later growth phases (9). In contrast to CatA, CatC, another catalase related protein, showed increased levels in all growth phases compared to the exponential phase. However, both catalases increased in the stationary phase, a pattern also seen in the proteome of another bacterium, *Shigella flexneri* (10). In *R. sphaeroides*, *catA*, but not *catC*, was shown to respond to hydrogen peroxide stress in an OxyR-dependent manner (11). For all of these proteins which decreased in the transition phase, their respective transcript levels did not decrease in this phase, with the single exception of *fbcB* (cytochrome b) whose transcript levels decreased in the transition phase.

**Changes in the stationary phase proteome**

In early stationary phase (28 h after inoculation), levels of 175 proteins increased by a log_2_ fold change ≥ 1, while 335 proteins increased by a log_2_ fold change ≥ 0.65 compared to exponential phase. These proteins belong primarily to COG E (amino acid transport and metabolism, 36 proteins), COG G (carbohydrate transport and metabolism, 23 proteins), COG C (energy production and conversion, 22 proteins including 4 genes of the *nuo* operon for NADH dehydrogenase), and COG P (inorganic transport and metabolism, 19 proteins). While the functions of many of these proteins are unknown, a scavenger-like scenario is conceivable, whereby the levels of transporters for carbohydrates, amino acids and inorganic molecules increase in response to the decreased availability of these molecules.

While the levels of most of these proteins stay high during deep stationary phase, some show decreased levels or were not detected at later stages. This is the case for BchP (RSP_0277), an enzyme involved in bacteriochlorophyll synthesis, and CrtE (RSP_0265), an enzyme involved in carotenoid synthesis. Other Bch proteins detected in the proteome analysis were already decreased in early stationary phase (BchE, BchN, BchZ) or even in transition phase (Bch B). Decreases in deep stationary phase were also observed for the hypothetical proteins RSP_7382, RSP_2046, and RSP_3825, for LytB (RSP_1666, a 4-hydroxy-3methylbut-2-enyl diphosphate reductase), ExoA (RSP_2563, a glycosyl transferase), FeoA (RSP_1819, the ferrous iron transporter), a glyoxylase (RSP_0799), and an antifreeze protein (RSP_0307). OmpW (RSP_2507), an outer membrane protein, also decreased in the stationary phase. In contrast, the OmpW of *Shigella flexneri* was the most abundant protein in the stationary phase cultures of this bacterium (10), possibly related to its role in evasion of host immunity (12).

Remarkably, in early stationary phase, the number of proteins with decreasing levels was less than the number of proteins with increasing levels. 127 proteins decreased by a log_2_ fold change of ≥ 1.0, and 301 proteins by a log_2_ fold change ≥ 0.65.

Deeper into stationary phase, the number of proteins with changed levels (log_2_ fold change of ≥ 1 compared to the exponential phase) grew (Fig. 3B). For example, at 28 h stationary phase, 175 proteins showed increased levels, while at 72 h and 144 h, the number of proteins with increased levels was 329 and 402, respectively. Likewise, the number of proteins with decreased levels (log_2_ fold change of ≤ -1) also increased with incubation time. 127 proteins were decreased at 28 h, 234 proteins at 72 h, and 333 proteins at 144 h. Extending the duration of the stationary phase also resulted in greater changes in the amount of protein. For example, RSP_3869 (the subunit of a manganese ABC transporter) showed a log_2_ fold change 5.4 (42 fold) increase at 28 h, which extended to 6.7 (>100 fold) at 144 h. Similarly, RSP_1016 (a small heat shock protein) increased from a log_2_ fold change of 2.2 (4.6 fold) at 28 h stationary phase to a log_2_ fold change of 8.9 (477 fold) and 8.55 (375 fold) at 72 h and 144 h, respectively. RSP_1016 is a member of the HSP20 family and the very high amounts of this chaperone are most likely required to protect the cellular proteins from misfolding and degradation upon prolonged stationary phase. RPS_1016 transcript levels increased after 72 h of incubation and in response to singlet oxygen in *R. sphaeroides* (13). Another member of the HSP20 family, RSP_1572, also increased after prolonged stationary phase, but only by a factor of 2. In contrast, other chaperone/chaperonins, GroEL and GroES, have similar levels in all growth phases.

Our analysis reveals that the proteome continues to change even in an extended stationary phase. Like RSP_1016, many other proteins increased only after a prolonged stationary phase. This category was based on having a log_2_ fold change ≤ 0.65 in early stationary phase (28 h of growth) and ≥ 1 after a prolonged stationary phase (72 h of growth). It consists of 104 proteins. 24 are transporters, mostly ABC transporter subunits (19 proteins). 6 of the ABC transporter subunits are polyamine transporter subunits (RSP_1539, 1882, 1886, 2141, 3337, 3515). One ABC polyamine transporter subunit, RSP_1884, showed increased levels only after 144 h of growth, while others (RSP_0161, 1883, 3397) were already increased at 28 h. The physiological role of polyamines in living cells is not clear. They are viewed as primordial stress-related molecules, which are induced by diverse stresses. They may aid survival by functioning as ROS scavengers, acid tolerance factors and chaperones and regulate stress responses (14, 15). Our previous work demonstrated that addition of spermidine to aerobic cultures of *R. sphaeroides* caused increased ROS (reactive oxygen species) levels (16). Singlet oxygen stress leads to reduced transcript levels of the *pot* genes (RSP_1882-1886) encoding a spermidine importer (13). Under stress conditions the sRNA SorX leads to downregulation of the Pot system, possibly to reduce oxidative stress (16). These data suggest that spermidine causes stress. On the other hand, our proteomic data has revealed a general up-regulation of spermidine import transporters in prolonged stationary phase, with the single exception of the ATPase subunit of a spermidine/putrescine transporter (RSP_0015). This situation argues for a role for spermidine in protection against stress. Interestingly, despite the increase of Pot proteins in the stationary phase, their respective transcript levels remain unchanged, suggesting that the up-regulation of Pot proteins is due to posttranscriptional events.

Other proteins that increased only in deep stationary phase (72 h or 144 h) include 8 ABC transporter subunits predicted to transport amino acids or peptides (RSP_1812, 2582, 2923, 3297, 3525, 3968) and glycine/betaine (RSP_2181, 3998). Furthermore, at least 9 predicted transcriptional regulators (RSP_0186, 0415, 0755, 0981, 1055, 1871, 2200, 2410, 2950) show a first significant increase at 72 h or 144 h incubation. This scenario strongly supports that the idea that at least some cells in deep stationary phase have not shut-down all activity but are actively regulating gene expression.

Clear decreases in the levels of 234 proteins (log_2_ fold change ≤ -1) can also been seen in the extended stationary phase. Two proteins with a remarkably strong decrease are involved in sulfur metabolism: RSP_1942 (sulfite/nitrite reductase) shows an 860 fold reduction, RSP_2738 (putative Rhodanese-related sulfurtransferase), a 560 fold reduction. Other proteins that decrease at this stage are involved in the photosynthetic apparatus. Here, the decrease in photosynthetic proteins generally correlates well with transcript changes.

A breakdown of the stationary phase regulated proteins into COGs reveals that the distribution between up and down regulated proteins is generally proportional (Fig. 4). One exception was COG G (carbohydrate transport and metabolisms) in which 24 proteins were increased but only 5 proteins were decreased. This suggests an active metabolic state, even at this late growth stage. However, the activity appears to be adapted to the stationary phase. For example, for COG E (amino acid transport and metabolism), 41 proteins decreased while only 17 increased. Likewise, only 2 proteins each from COG L (replication, recombination, repair) and J (translation, ribosome structure and biogenesis) were increased, while 15 proteins were decreased in COG L and 12 in COG J, indicating that synthesis of DNA is low and that production of proteins was more specialized. Furthermore, decreases in 19 proteins of COG M (cell envelope synthesis) fit with the cessation of growth and cell division. However, unexpected increases in 12 proteins of COG M at this late stage underscore the fact that regulation of cell division-related proteins during the stationary phase is still poorly understood in *R. sphaeroides*.

Proteomic changes (log_2_ fold change ≥ 1) specific to deep stationary phase (144 h), apparent from a comparison between levels at 72 h and 144 h, included 88 proteins. These are distributed among almost all COGs, most of which belong to COG E (amino acid transport and metabolism). As expected, the protein changes in COG F (nucleotide transport and metabolism) and J (translation, ribosome biogenesis) were mostly down, indicating that these processes are less active in deep stationary phase.

**Changes in protein levels during outgrowth**

Long-term survival of bacterial populations requires the ability to resume growth again after periods at stationary phase. The COG-based analysis clearly indicates that the protein content changes very little during the outgrowth from stationary phase (Fig. 4). As expected, the 90 minutes of outgrowth is not sufficient to allow for a major remodeling of the proteome. As we noted above, this is not the case with the transcriptome, which undergoes marked changes after 90 minutes, many of which are apparent even at 20 minutes (see PCC values in Tables 2A & 2B). However, the fact that some proteins were rapidly changed in abundance during outgrowth demonstrates this capacity. For example, a strong increase (92 fold) was observed for a ferrisiderophore (RSP_1440) after 90 minutes of outgrowth following 28 h stationary phase. Other proteins increasing in the outgrowth include a monoxygenase involved in K^+^ transport (RSP_3323) and an ABC Fe^3+^ siderophore transporter (RSP_3079). We speculate that this pattern indicates a high demand for inorganic ions, especially iron, during the outgrowth.

Interestingly all 4 subunits of the carbon monooxygenase (RSP_2876-2879) showed a sudden increase in the outgrowth following 28 h stationary phase. Carbon monoxygenase is involved in the glutathione-dependent detoxification of formaldehyde that forms during oxidative protein carbonylation through ROS. The genes for these proteins are part of an operon, which is negatively (and indirectly) influenced by the sRNAs Ccsr1-4 upon oxidative stress. It has been suggested that the CcsR1-4 help to control the allocation of glutathione to different pathways of oxidative stress defense (Billenkamp et al., 2016).

Despite the strong overall similarity in protein accumulation between the stationary phase and the following outgrowth, proteins of COG M (cell wall, membrane, envelope synthesis) and COG T (signal transduction) provide an exception. Plots of COG M and T (Fig. 4) indicate significant differences in protein changes between the 28 h stationary phase and the following outgrowth. This pattern fits with the expectation that proteins involved in cell division and signaling constitute the bulk of proteins necessary for the bacteria to detect the environmental change (i.e., fresh nutrients) and to respond with cell wall synthesis. Protein changes in the outgrowth from extended stationary phase (72 h and 144 h) are less dramatic, indicating that the outgrowth response is slower with increase age of stationary phase cultures.

**References**

1. Zong R, Jiao N. Proteomic responses of *Roseobacter litoralis* OCh149 to starvation and light regimen. Microbes Environ. 2012;27(4):430-42.

2. Newman JD, Falkowski MJ, Schilke BA, Anthony LC, Donohue TJ. The *Rhodobacter sphaeroides* ECF sigma factor, sigma(E), and the target promoters *cycA* P3 and *rpoE* P1. J Mol Biol. 1999;294(2):307-20.

3. Nuss AM, Adnan F, Weber L, Berghoff BA, Glaeser J, Klug G. DegS and RseP homologous proteases are involved in singlet oxygen dependent activation of RpoE in *Rhodobacter sphaeroides*. PloS one. 2013;8(11):e79520.

4. Anthony JR, Newman JD, Donohue TJ. Interactions between the *Rhodobacter sphaeroides* ECF sigma factor, sigma(E), and its anti-sigma factor, ChrR. J Mol Biol. 2004;341(2):345-60.

5. Glaeser J, Zobawa M, Lottspeich F, Klug G. Protein synthesis patterns reveal a complex regulatory response to singlet oxygen in *Rhodobacter*. Journal of proteome research. 2007;6(7):2460-71.

6. Anthony JR, Warczak KL, Donohue TJ. A transcriptional response to singlet oxygen, a toxic byproduct of photosynthesis. Proc Natl Acad Sci U S A. 2005;102(18):6502-7.

7. Gough SP, Petersen BO, Duus JO. Anaerobic chlorophyll isocyclic ring formation in *Rhodobacter capsulatus* requires a cobalamin cofactor. Proc Natl Acad Sci U S A. 2000;97(12):6908-13.

8. Zappa S, Li K, Bauer CE. The tetrapyrrole biosynthetic pathway and its regulation in *Rhodobacter capsulatus*. Advances in experimental medicine and biology. 2010;675:229-50.

9. Remes B, Rische-Grahl T, Muller KMH, Forstner KU, Yu SH, Weber L, et al. An RpoHI-Dependent Response Promotes Outgrowth after Extended Stationary Phase in the Alphaproteobacterium *Rhodobacter sphaeroides*. Journal of bacteriology. 2017;199(14).

10. Zhu L, Liu XK, Zhao G, Zhi YD, Bu X, Ying TY, et al. Dynamic proteome changes of *Shigella flexneri* 2a during transition from exponential growth to stationary phase. Genomics, proteomics & bioinformatics. 2007;5(2):111-20.

11. Zeller T, Klug G. Detoxification of hydrogen peroxide and expression of catalase genes in *Rhodobacter*. Microbiology. 2004;150(Pt 10):3451-62.

12. Li W, Wen L, Li C, Chen R, Ye Z, Zhao J, et al. Contribution of the outer membrane protein OmpW in *Escherichia coli* to complement resistance from binding to factor H. Microb Pathog. 2016;98:57-62.

13. Berghoff BA, Konzer A, Mank NN, Looso M, Rische T, Forstner KU, et al. Integrative "omics"-approach discovers dynamic and regulatory features of bacterial stress responses. PLoS genetics. 2013;9(6):e1003576.

14. Yoshida M, Kashiwagi K, Shigemasa A, Taniguchi S, Yamamoto K, Makinoshima H, et al. A unifying model for the role of polyamines in bacterial cell growth, the polyamine modulon. The Journal of biological chemistry. 2004;279(44):46008-13.

15. Rhee HJ, Kim EJ, Lee JK. Physiological polyamines: simple primordial stress molecules. J Cell Mol Med. 2007;11(4):685-703.

16. Peng T, Berghoff BA, Oh JI, Weber L, Schirmer J, Schwarz J, et al. Regulation of a polyamine transporter by the conserved 3' UTR-derived sRNA SorX confers resistance to singlet oxygen and organic hydroperoxides in *Rhodobacter sphaeroides*. RNA biology. 2016;13(10):988-99.

**Figure legends**

Fig. S1: Heatmaps of Pearson´s correlation coefficients for the transcriptome (A) and the proteome (B) and Spearman correlation between the transcriptome and proteome samples (C). The degree of correlation between any two conditions is indicated by the coloring. For A & B, the matrix diagonal serves as the symmetry axis, and dendrograms indicate similarity between growth phases.

Fig. S2: Scatter plots (A) and heat maps (B) comparing changes of the transcriptome and the proteome in different phases of growth. The scatterplots are divided into distinct areas to distinguish between groups of proteins that are, for instance, equally increased in both RNA and protein levels (right part of the dashed diagonal lines) or decreased on the RNA level but not at the protein level (left part of the dashed horizontal lines). Regulation was defined as the detection of ≥2-fold change, equivalent to a log2 fold change higher than 1 or lower than -1, respectively. Each of the genes found in the respective groups are listed in Table S4.
