# Supplementary material for: Comparative analyses of the variation of the transcriptome and proteome of Rhodobacter sphaeroides throughout growth
Source: BMC Genomics. 2019 May 9;20:358. doi: 10.1186/s12864-019-5749-3 (PMC6509803; doi:10.1186/s12864-019-5749-3)
Supplement: Supplementary file 5 — Figure S1. Heatmaps of correlations between transcriptome and proteome. (PPTX 317 kb) [file 12864_2019_5749_MOESM5_ESM.pptx]

## Slide 1
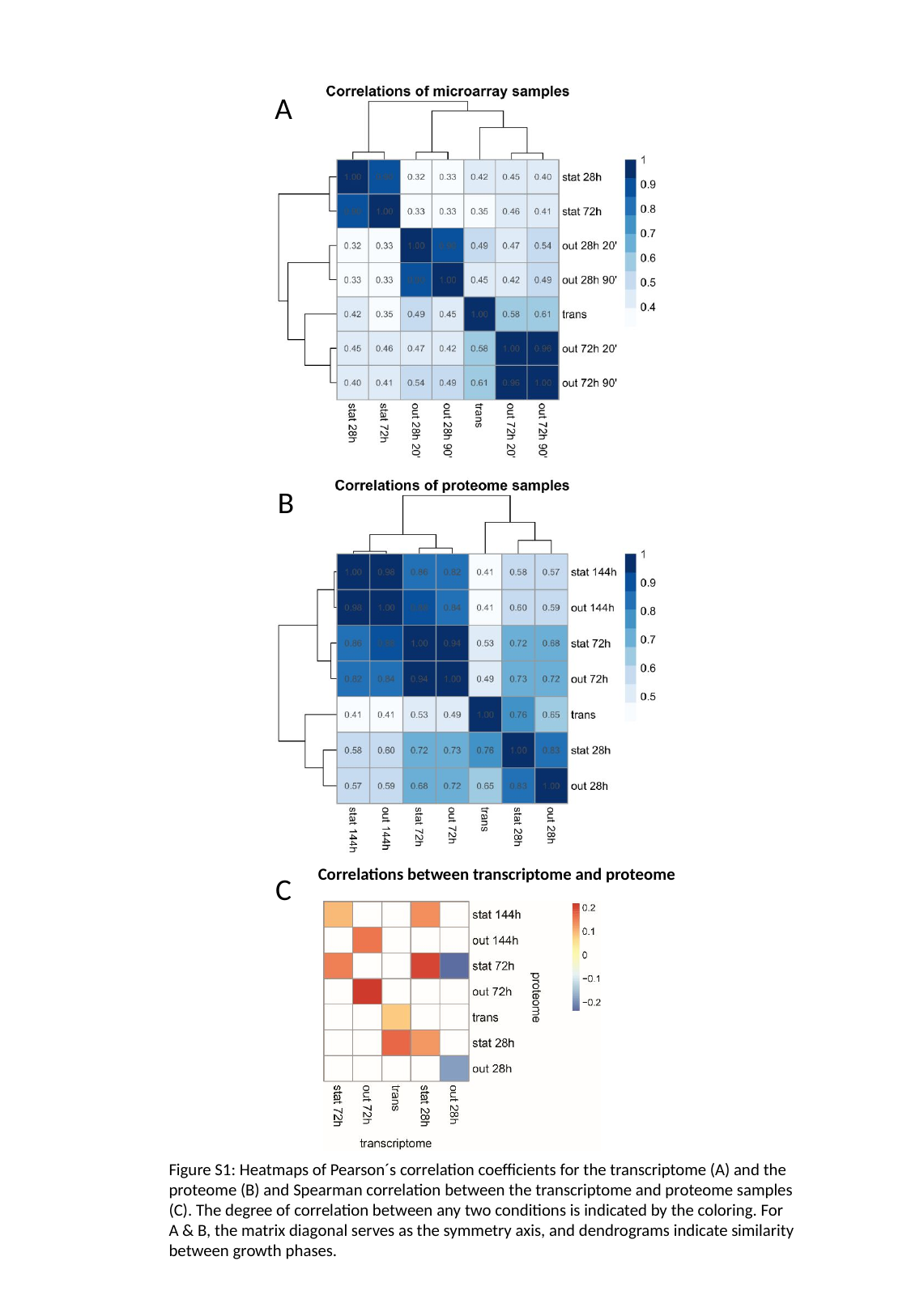

A
B
Correlations between transcriptome and proteome
C
Figure S1: Heatmaps of Pearson´s correlation coefficients for the transcriptome (A) and the proteome (B) and Spearman correlation between the transcriptome and proteome samples (C). The degree of correlation between any two conditions is indicated by the coloring. For A & B, the matrix diagonal serves as the symmetry axis, and dendrograms indicate similarity between growth phases.
