# Supplementary material for: Comparative analyses of the variation of the transcriptome and proteome of Rhodobacter sphaeroides throughout growth
Source: BMC Genomics. 2019 May 9;20:358. doi: 10.1186/s12864-019-5749-3 (PMC6509803; doi:10.1186/s12864-019-5749-3)
Supplement: Supplementary file 6 — Figures S2A-K. Scatter plots of transcriptome versus proteome throughout growth phases. (PPTX 1040 kb) [file 12864_2019_5749_MOESM6_ESM.pptx]

## Slide 1
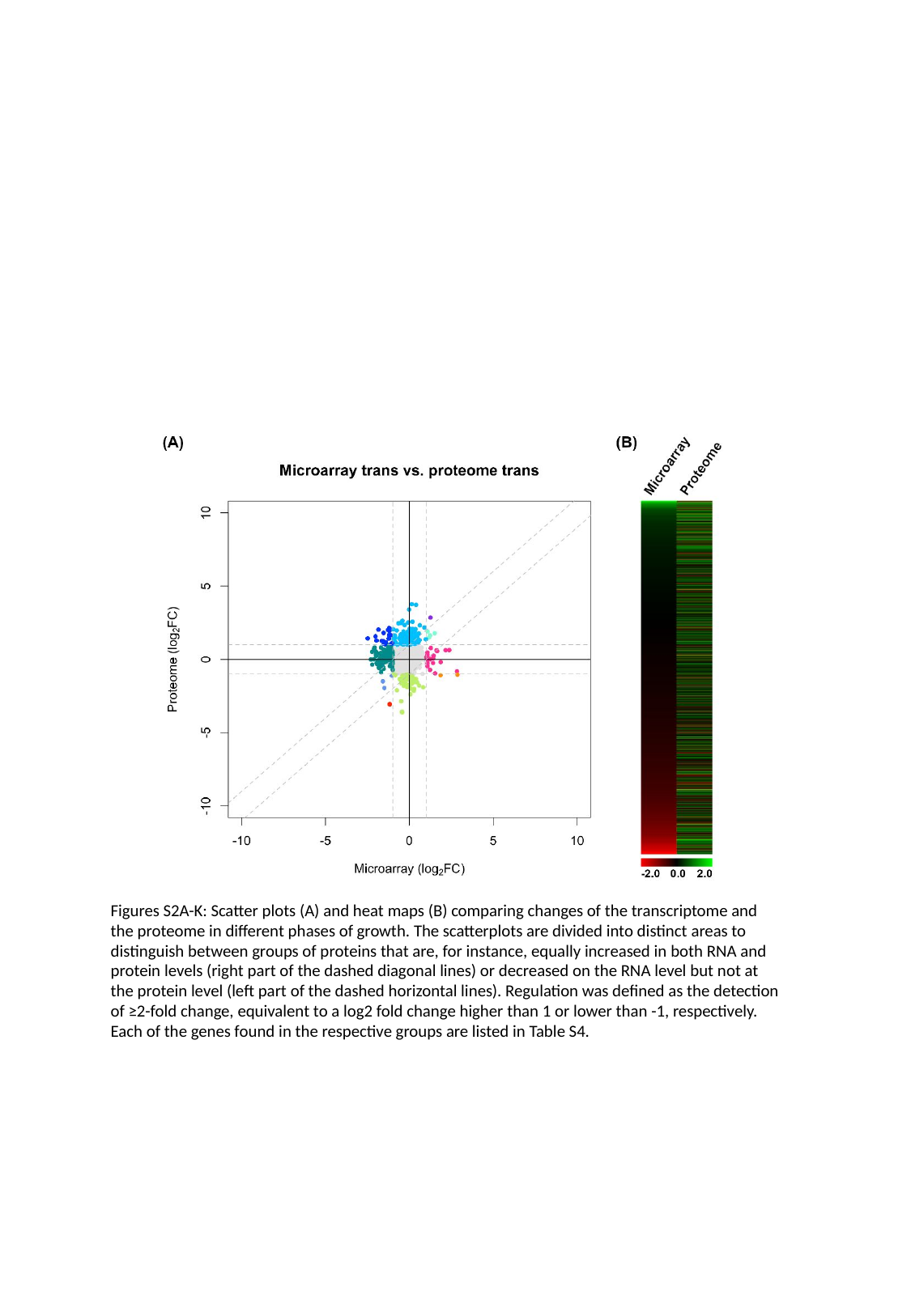

Figures S2A-K: Scatter plots (A) and heat maps (B) comparing changes of the transcriptome and the proteome in different phases of growth. The scatterplots are divided into distinct areas to distinguish between groups of proteins that are, for instance, equally increased in both RNA and protein levels (right part of the dashed diagonal lines) or decreased on the RNA level but not at the protein level (left part of the dashed horizontal lines). Regulation was defined as the detection of ≥2-fold change, equivalent to a log2 fold change higher than 1 or lower than -1, respectively. Each of the genes found in the respective groups are listed in Table S4.

## Slide 2
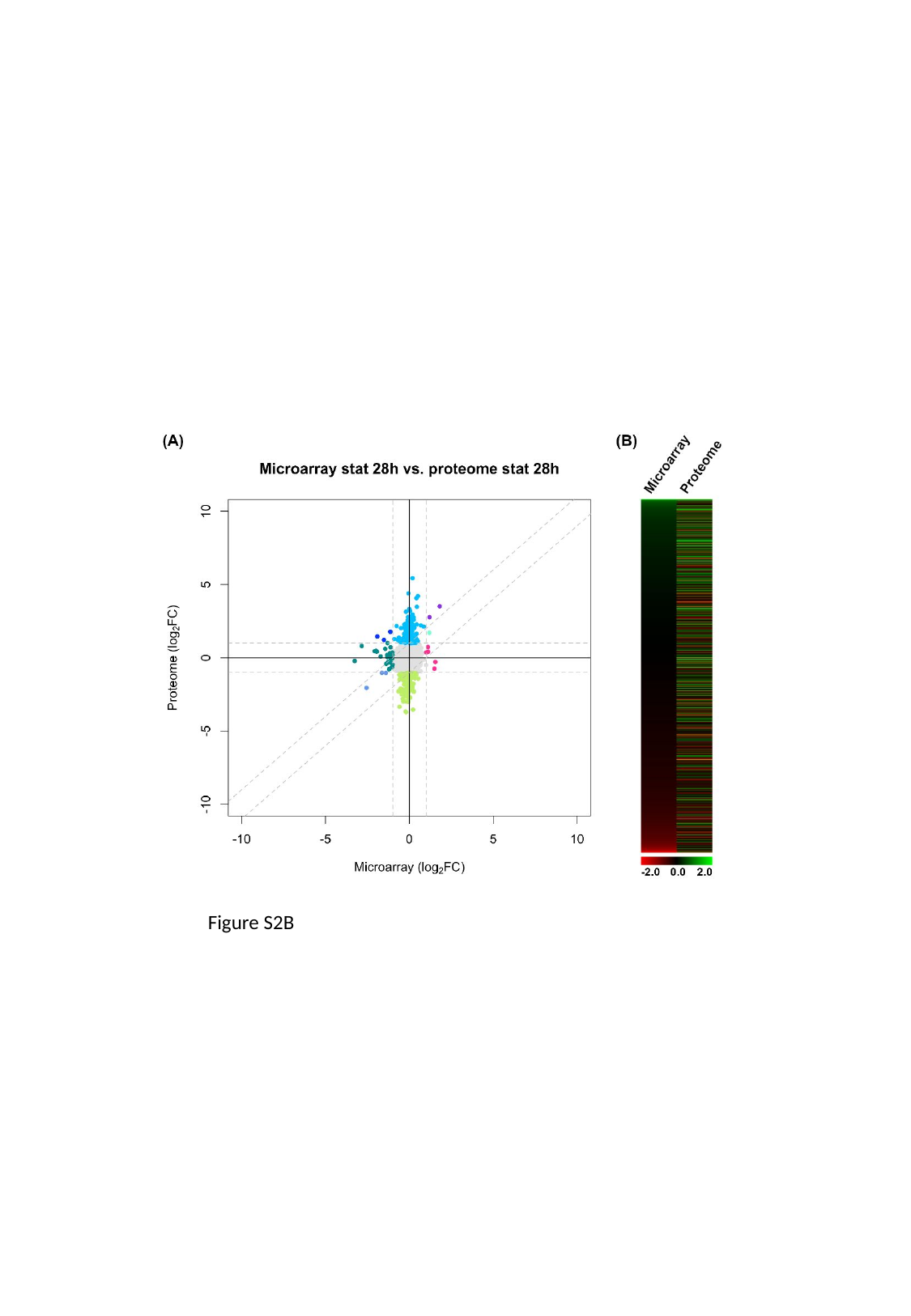

Figure S2B

## Slide 3
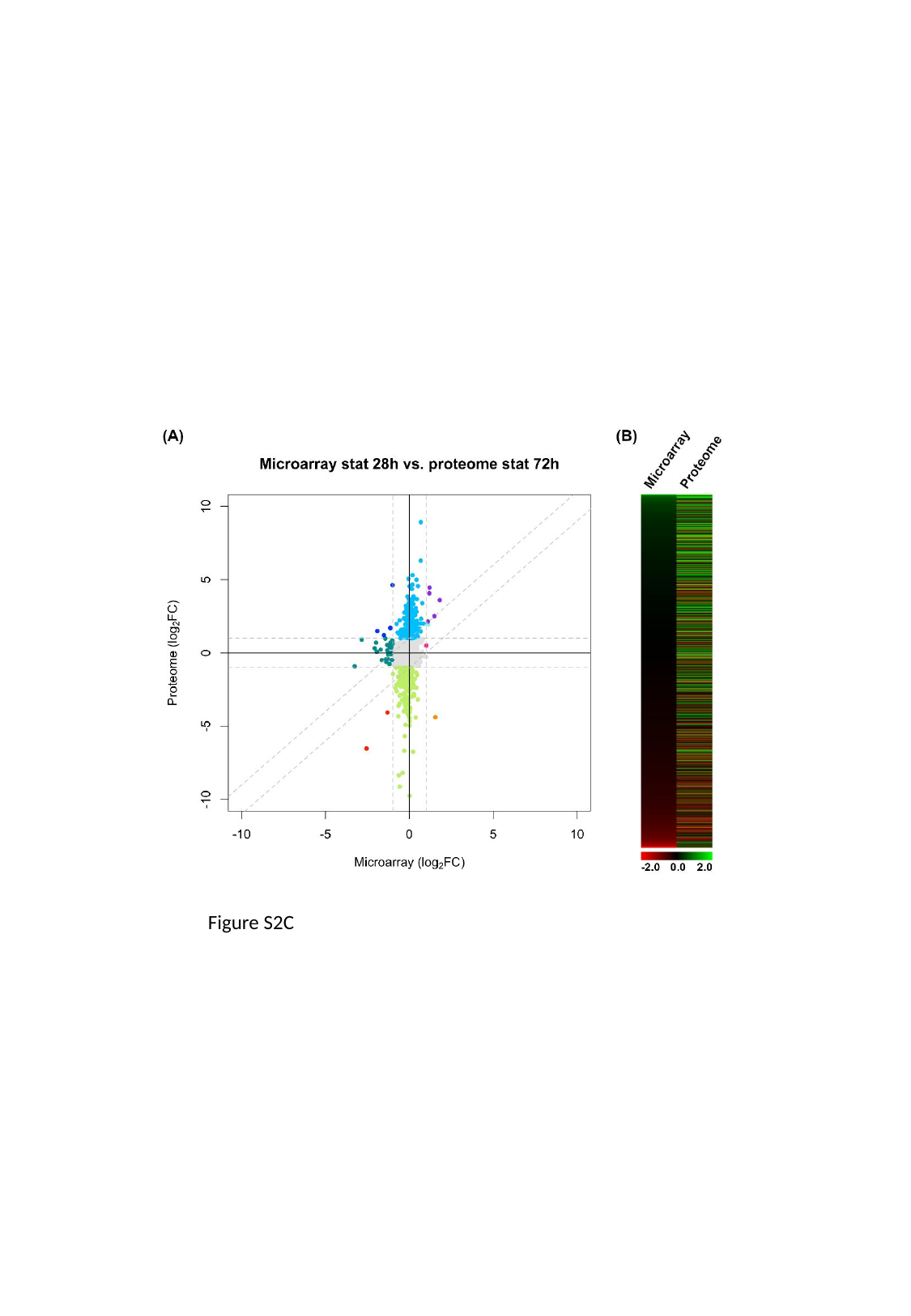

Figure S2C

## Slide 4
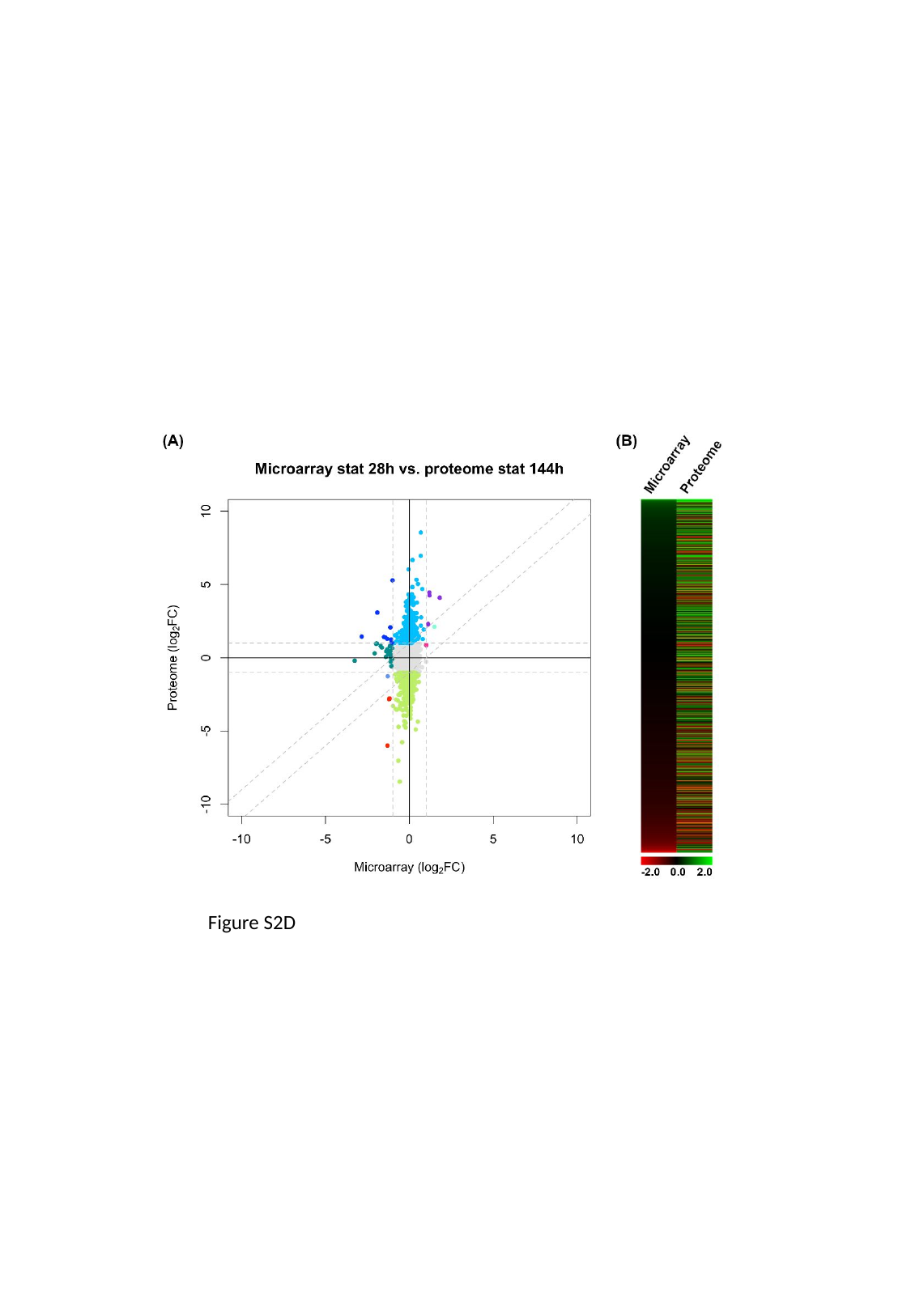

Figure S2D

## Slide 5
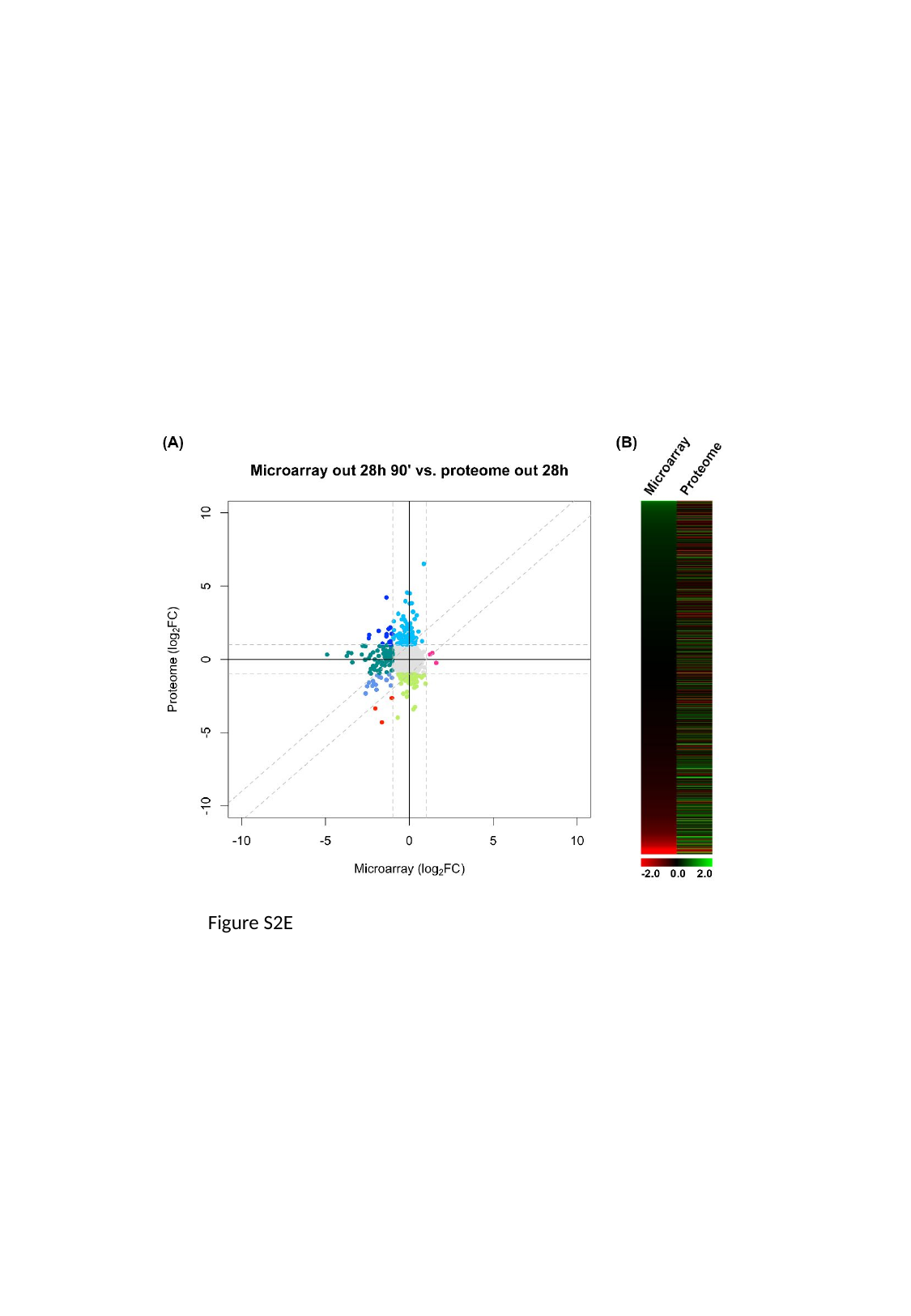

Figure S2E

## Slide 6
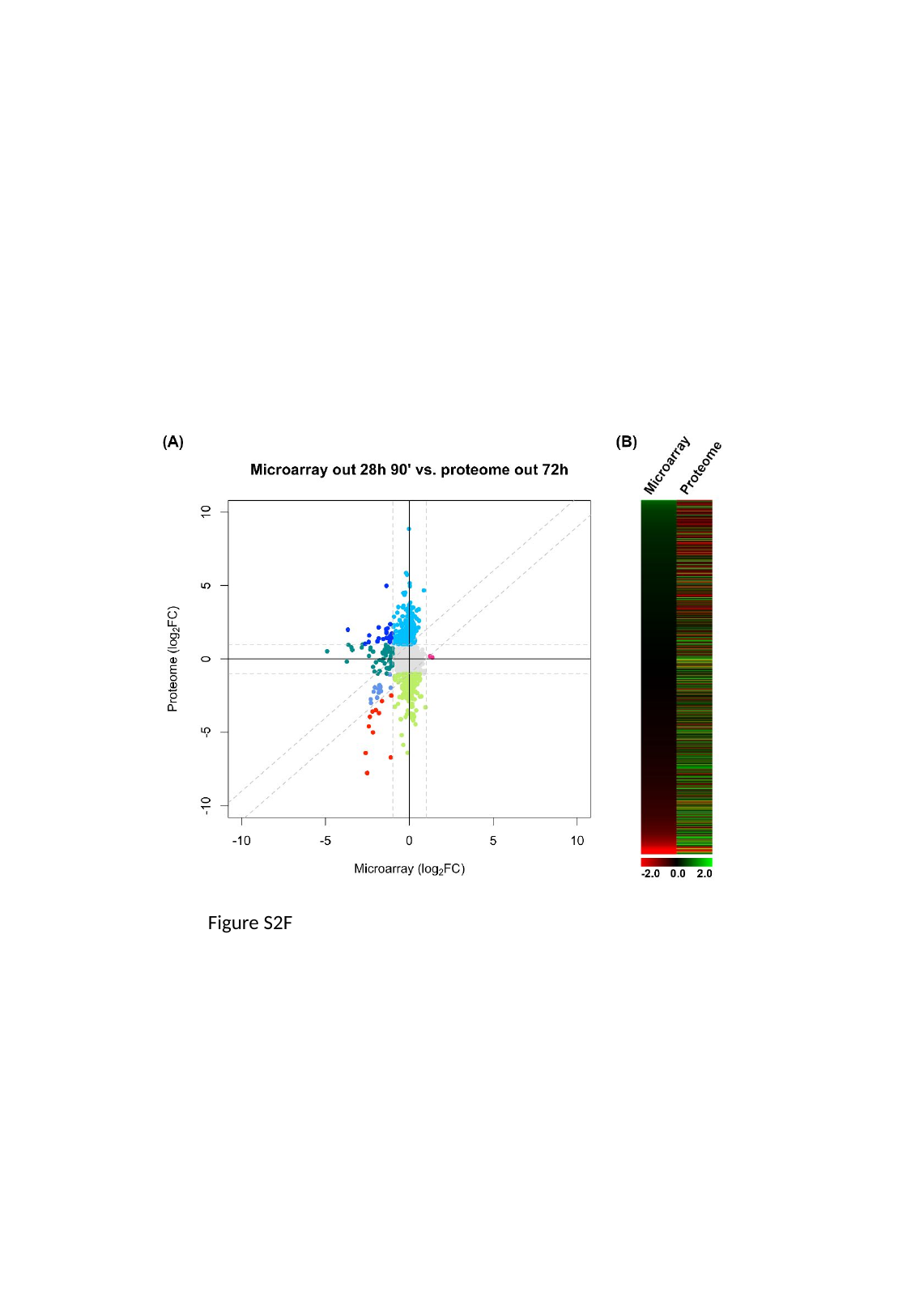

Figure S2F

## Slide 7
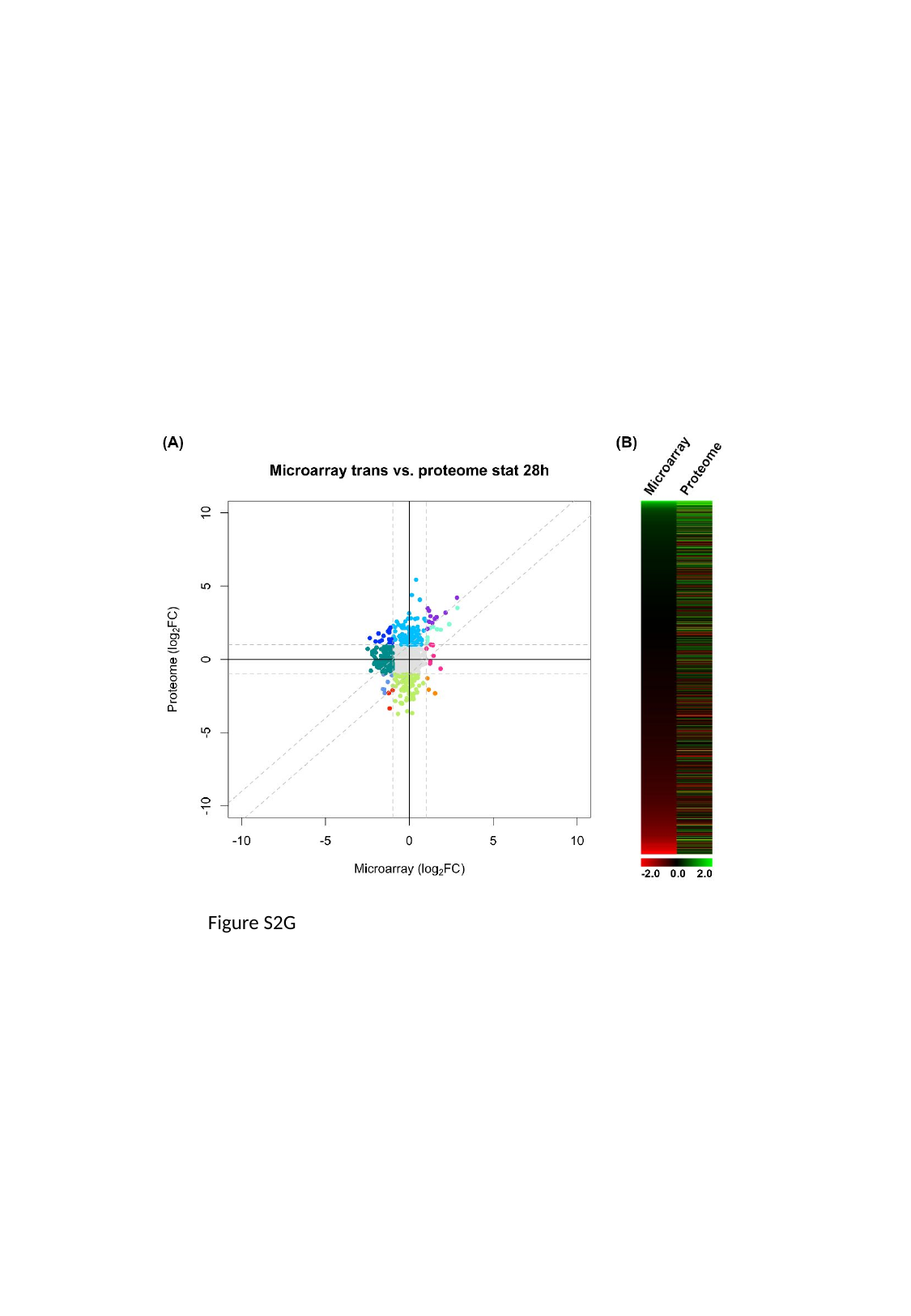

Figure S2G

## Slide 8
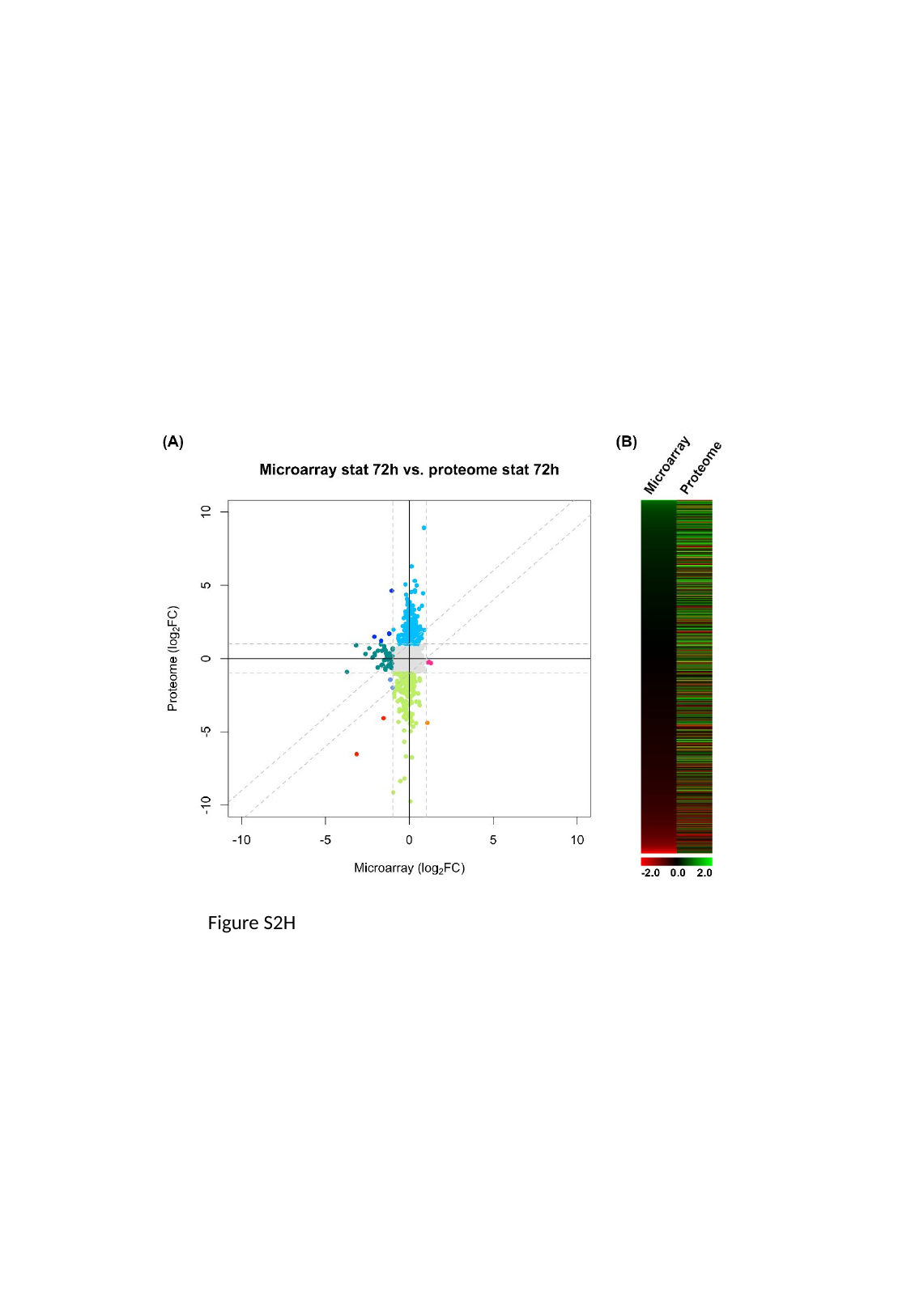

Figure S2H

## Slide 9
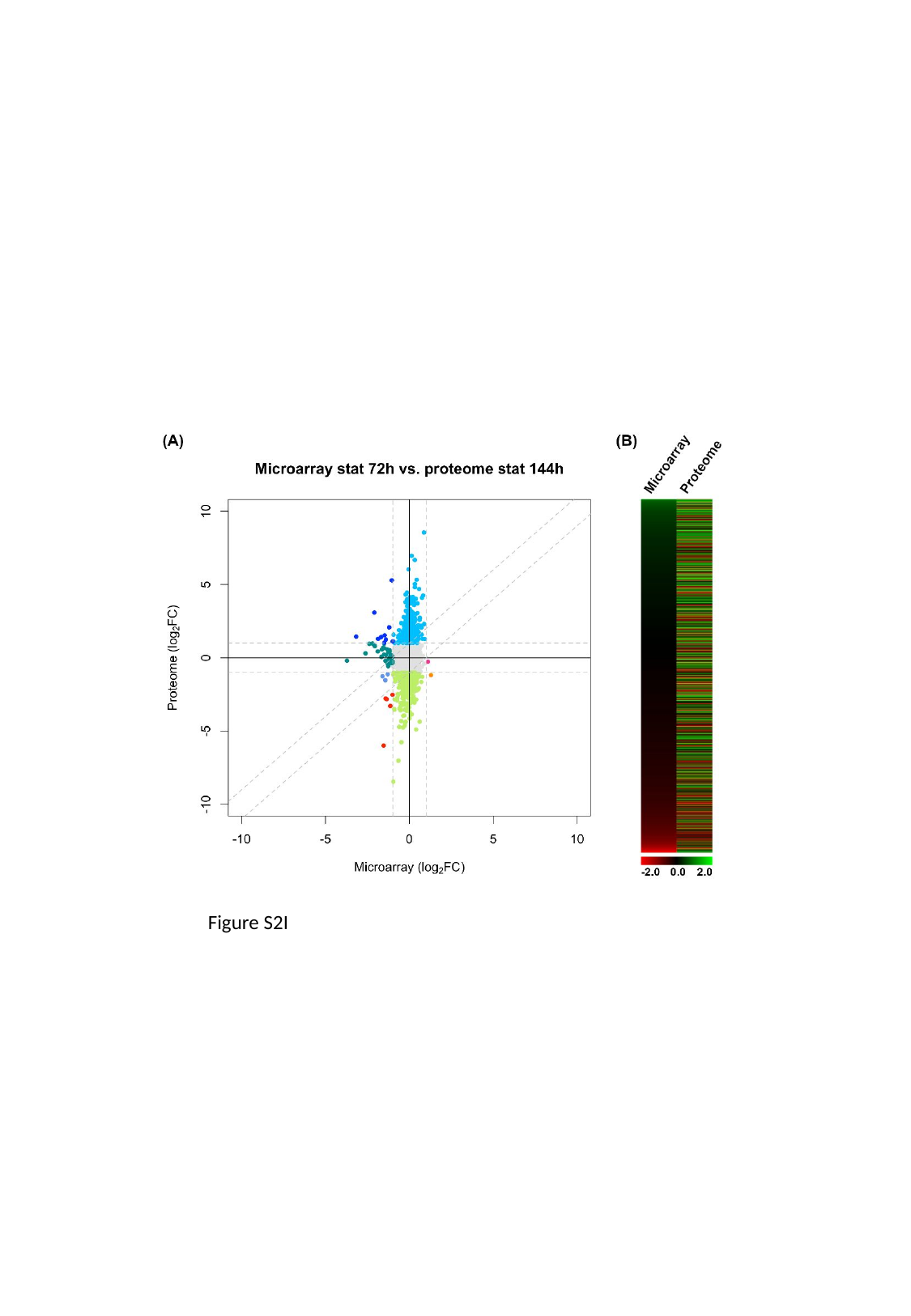

Figure S2I

## Slide 10
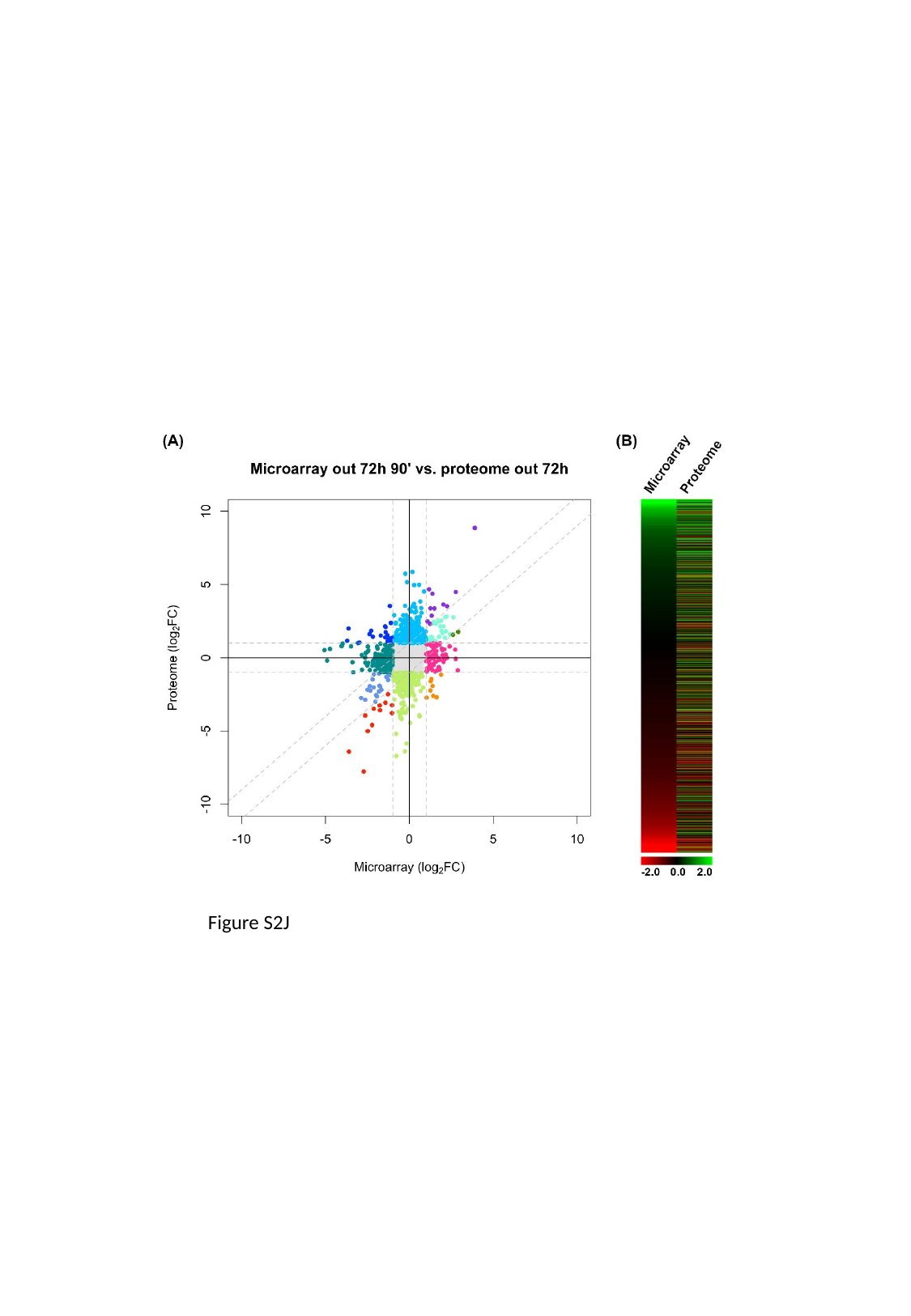

Figure S2J

## Slide 11
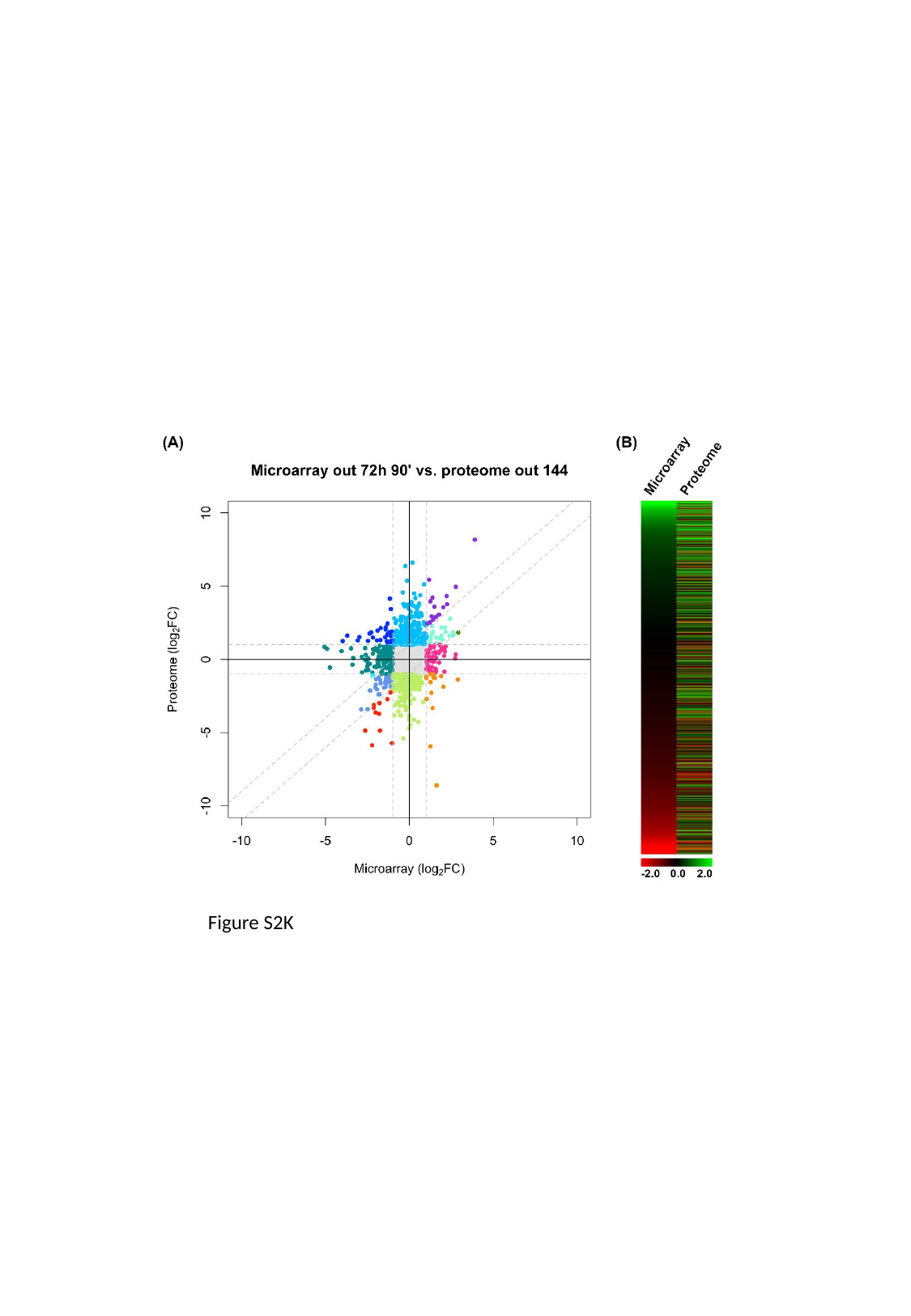

Figure S2K
